# Wearable activity monitors to assess performance status and predict clinical outcomes in advanced cancer patients

**DOI:** 10.1038/s41746-018-0032-6

**Published:** 2018-07-05

**Authors:** Gillian Gresham, Andrew E. Hendifar, Brennan Spiegel, Elad Neeman, Richard Tuli, B. J. Rimel, Robert A. Figlin, Curtis L. Meinert, Steven Piantadosi, Arvind M. Shinde

**Affiliations:** 1 Samuel Oschin Comprehensive Cancer Institute, Cedars-Sinai Medical Center, Los Angeles, CA USA; 2 Department of Epidemiology, Johns Hopkins Bloomberg School of Public Health, Baltimore, MD USA; 3 Department of Health Services Research, Cedars-Sinai Health System, Cedars-Sinai Medical Center, Los Angeles, CA USA

**Keywords:** Cancer, Quality of life, Outcomes research

## Abstract

An objective evaluation of patient performance status (PS) is difficult because patients spend the majority of their time outside of the clinic, self-report to providers, and undergo dynamic changes throughout their treatment experience. Real-time, objective activity data may allow for a more accurate assessment of PS and physical function, while reducing the subjectivity and bias associated with current assessments. Consenting patients with advanced cancer wore a wearble activity monitor for three consecutive visits in a prospective, single-cohort clinical trial. Provider-assessed PS (ECOG/Karnofsky) and NIH PROMIS® patient-reported outcomes (PROs) were assessed at each visit. Associations between wearable activity monitor metrics (steps, distance, stairs) and PS, clinical outcomes (adverse events, hospitalizations, survival), and PROs were assessed using correlation statistics and in multivariable logistic regression models. Thirty-seven patients were evaluated (54% male, median 62 years). Patients averaged 3700 steps, 1.7 miles, and 3 flights of stairs per day. Highest correlations were observed between average daily steps and ECOG-PS and KPS (*r* *=* 0.63 and *r* *=* 0.69, respectively *p* < 0.01). Each 1000 steps/day increase was associated with reduced odds for adverse events (OR: 0.34, 95% CI 0.13, 0.94), hospitalizations (OR: 0.21 95% CI 0.56, 0.79), and hazard for death (HR: 0.48 95% CI 0.28–0.83). Significant correlations were also observed between activity metrics and PROs. Our trial demonstrates the feasibility of using wearable activity monitors to assess PS in advanced cancer patients and suggests their potential use to predict clinical and patient-reported outcomes. These findings should be validated in larger, randomized trials.

## Wearable tech: Activity monitor tracks wellbeing of advanced cancer patients

Wearable activity monitors provide an objective and continuous measure of general wellbeing and physical function among patients with advanced cancer. Gillian Gresham from Cedars-Sinai Medical Center in Los Angeles, California, USA, and colleagues enlisted 37 patients with metastatic or inoperable cancer to wear wristbands that wireless measure heart rate and activity patterns over the span of 2 weeks. At weekly clinic visits, the patients also filled in health questionnaires and their healthcare providers made independent evaluations. The researchers found a reasonably high correlation between the clinician-assessed performance status and the average daily step total calculated by the wristband. More steps also translated into lower odds of serious complications, hospitalizations or death. The study shows the feasibility of using wearable technology to track the ability of patients with advanced cancer to perform certain activities of daily living.

## Introduction

Cancer patients require an accurate assessment of performance status and physical function to inform treatment decisions and determine eligibility into clinical trials. However, an objective evaluation of physical function and performance status is difficult because patients spend the majority of their time outside of the clinic, self-report to providers, and undergo dynamic changes throughout their treatment experience.

The Eastern Cooperative Oncology Group (ECOG) Performance Status (PS) (1960) and the Karnofsky Performance Status (KPS) (1949) are common scales used for assessing a patient’s level of function and ability of self-care.^1,2^ Despite their routine use and value in oncology, there are several limitations associated with these scales. First, these scales are subjective and physician-reported, thus leading to the potential for under- or over-estimation of a patient’s performance status.^3–5^ For instance, a physician’s intent to provide therapy or enroll a patient into a clinical trial may inadvertently result in overestimation of physical function and can further increase the patient’s risk for toxicities and treatment intolerance.^5^ Conversely, an underestimation of physical robustness may result in under treatment, which can subsequently affect a patient’s clinical outcomes and quality of life. Second, performance status assessment is limited by recall bias where patients may not have a complete recollection of their past activity and symptoms, which may be used to inform performance status ratings.^4^ Recall may also be influenced by a patient’s desire to enroll in a trial or receive therapy, thus resulting in the over reporting of their physical activity levels and function. A third challenge is the static nature of performance status assessment, where ECOG-PS and KPS are only captured periodically during clinic visits. However, a patient’s performance status is dynamic over the course of treatment and can change on a daily basis.^3^ Finally, the quality and consistency of performance status reporting in oncology is suboptimal and there can be disagreements in performance status ratings between providers and nurses.^5–9^

Patient-reported outcomes (PROs) offer additional insight into a patient’s daily activity, and have been shown to have a prognostic and therapeutic value.^9^ The NIH PROMIS® scales are increasingly recognized in oncology as capturing a broad range of relevant outcomes that may not be recorded in the medical chart.^9–12^ Thus, the routine collection of PROs has become an important component to the assessment of a patient's treatment experience.^9^ However, as with all self-reported outcomes, PROs are associated with the same response and recall biases and may introduce additional burden related to the frequent administration and completion of the questionnaires.^3,4^ Given the prognostic importance of performance status and PROs in addition to their impact on treatment decisions, there is a need for feasible, real-time, objective collection of a patient’s daily activity.^3,4^

Recent technological advances in wearable activity monitors have made it possible to collect real-time, objective patient activity data in a non-obtrusive manner. Wearable activity monitors measure the duration, intensity, and frequency of physical activity and have previously been used in clinical settings to motivate exercise and behavior.^13–15^ Consumer-based wearable activity monitors, such as the Fitbit Charge HR® are relatively inexpensive, simple devices that can be used to track physical activity including step counts, stairs climbed, calories, heart rate, and sleep.^16–18^ These will be referred to as *activity metrics* in this report. While previous studies have used wearable activity monitors for measuring adherence to a particular exercise intervention, or to motivate physical activity, their application for the assessment of patient functional and clinical outcomes has not previously been reported.^15^

Our primary objective was to measure the association between Fitbit Charge HR® activity metrics and performance status. We also sought to measure the association between the wearable activity metrics and survival, the occurrence of serious adverse events, as well as the correlation between the wearable activity metrics and PROs in the domains of pain, physical functioning, and fatigue.

## Results

### Patient characteristics

Thirty-seven patients consented to the study. There were 20 males and 17 females with a median age of 62 years (range 34–81) (Table 1). At baseline, patients had provider-assessed ECOG-PS scores of 0 (24%), 1 (35%), 2 (24%), or 3 (16%). The majority of patients were diagnosed with gastrointestinal malignancies (*n* = 27) and had stage 4 disease (*n* = 34). There were two patients with locally advanced stage 3 pancreatic disease and one patient with stage 3B endocervical serous carcinoma. Compared to the standard cancer population (mean 50, SD: 10), patients in this study had lower levels of physical functioning by approximately one standard deviation. Patients also reported higher levels of pain and fatigue compared to the standard cancer population. Fatigue, pain, and depression scores increased and reported sleep quality and physical functioning decreased as ECOG-PS increased. Additional patient baseline information is provided in Table 1.

**Table 1:** Demographic and clinical characteristics at baseline visit

Characteristics	*N* (%)
*Gender*	
Male	20 (45.9)
Female	17 (46.1)
*Age*	
Median (range)	62 (34, 81)
*Ethnicity*	
Hispanic	5 (13.5)
Non-Hispanic	32 (86.5)
*Race*	
Caucasian	23 (62.2)
Asian	7 (18.9)
Other	7 (18.9)
*Cancer type*	
Pancreas	27 (73.0)
Colorectal	2 (5.4)
Other gastrointestinal	5 (13.5)
Gynecological	2 (5.4)
Lung	1 (2.7)
*Cancer stage*	
4	34 (91.9)
3^a^	3 (8.1)
*Occupation*	
Currently working	18 (48.7)
Retired	8 (21.6)
Other	11 (29.7)
*Smoking status* ^b^	
Ever	10 (30.3)
Never	23 (69.7)
*ECOG*	
0	9 (24.3)
1	13 (35.1)
2	9 (24.3)
3	6 (16.2)
>3	0 (0.0)
*KPS*	
100	6 (16.2)
90	5 (13.5)
80	9 (24.3)
70	8 (21.6)
60	3 (8.1)
50	5 (13.5)
<50	1 (2.7)
*NIH PROMIS®*	
*Domain, mean (SD)*	
Physical functioning	41.5 (9.1)
Pain	56.9 (10.5)
Fatigue	57.2 (10.7)
Sleep	53.6 (8.4)
Depression	40.5 (10.2)

^a^Patients diagnosed with locally advanced pancreatic cancer (*n* = 2) and borderline resectable and stage 3B endocervical serous carcinoma (*n* = 1)

^b^Smoking status unknown in four patients

### Fitbit Charge HR® activity data are correlated with performance status

**Fig. 1:**
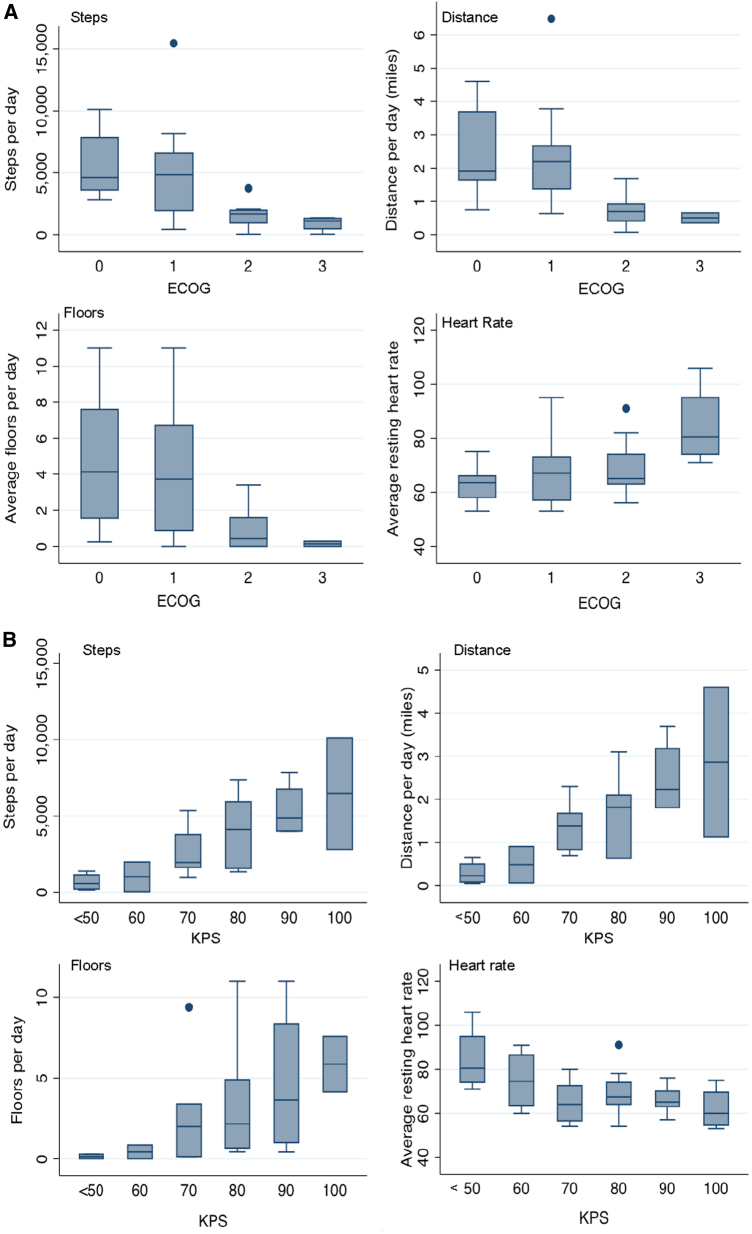
Fitbit Charge HR® activity metrics by **a** ECOG performance status and **b** Karnofsky performance status

On average, patients walked approximately 3700 steps, or 1.7 miles, per day, climbed three flights of stairs per day, and slept 8 h/night as measured with the wearable activity monitor. Average resting heart rate was 68 beats per minute. Average daily step counts were significantly different across ECOG-PS categories: ECOG-PS 0, 5345 steps; ECOG-PS 1, 4835 steps, ECOG-PS 2, 1553 steps, ECOG-PS 3, 902 steps (*p* < 0.0001). Activity metrics were correlated with ECOG-PS and KPS in the expected direction and magnitude: as ECOG-PS increased from 0 (good PS) to 3–4 (poor PS), average daily steps, distance, and stairs decreased (Fig. 1a). An inverse relationship was observed between KPS and average daily physical activity (Fig. 1b). The largest correlation coefficients (*r)* were observed between average steps per day and increasing ECOG-PS (*r* *=* 0.63, *p* < 0.01) and KPS (*r* *=* 0.69*, p* *<* 0.01). Statistically significant correlations were also observed between average distance travelled, stairs climbed, resting heart rate and both ECOG-PS and KPS. No significant correlation between device-assessed sleep duration and performance status was found.

**Fig. 2:**
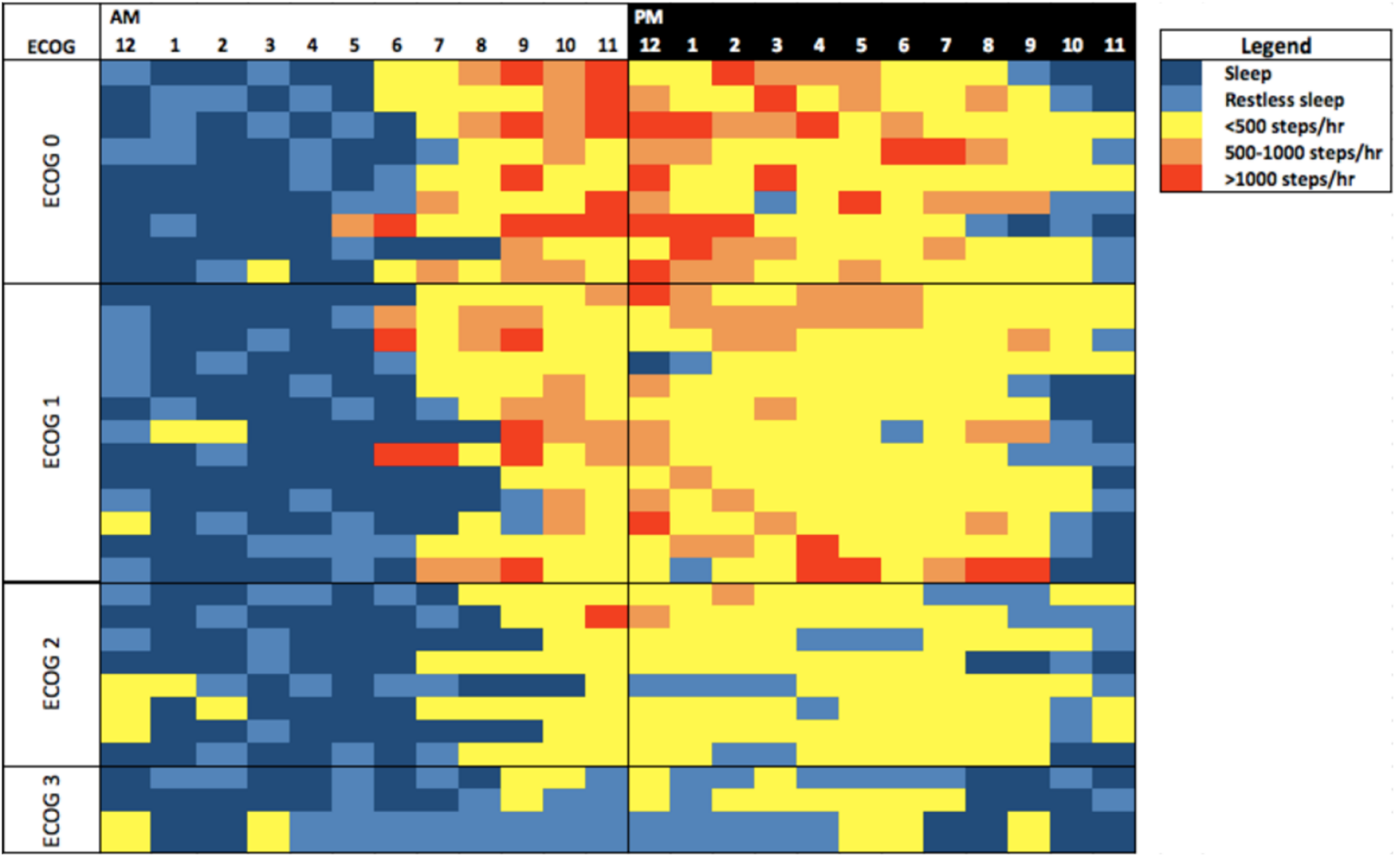
Heat map of average activity intensity for each patient over a 24 h period, as measured using the wearable activity monitor and sorted by ECOG-PS categories

Average patient activity over a 24 h period, sorted by ECOG-PS, is displayed in Fig. 2. The heat map provides a visual account of the variation in the average hourly activity of each patient, including sleep and physical activity, where the intensity and frequency of physical activity decreases as ECOG-PS worsens.

### Average daily step counts are independently associated with clinical outcomes

There were 11 deaths that occurred within 6-month follow-up. There were 35 treatment-related grade 3+ adverse events occurring in 13 patients (35%) within 30 days from the patient’s end-of-study visit (Supplementary Table 3). Most adverse events and hospitalizations occurred as a result of progressive disease, treatment side-effects, or other disease-related symptoms. One patient reported a mild rash on the wrist possibly related to the study intervention (wearable activity monitor).

**Fig. 3:**
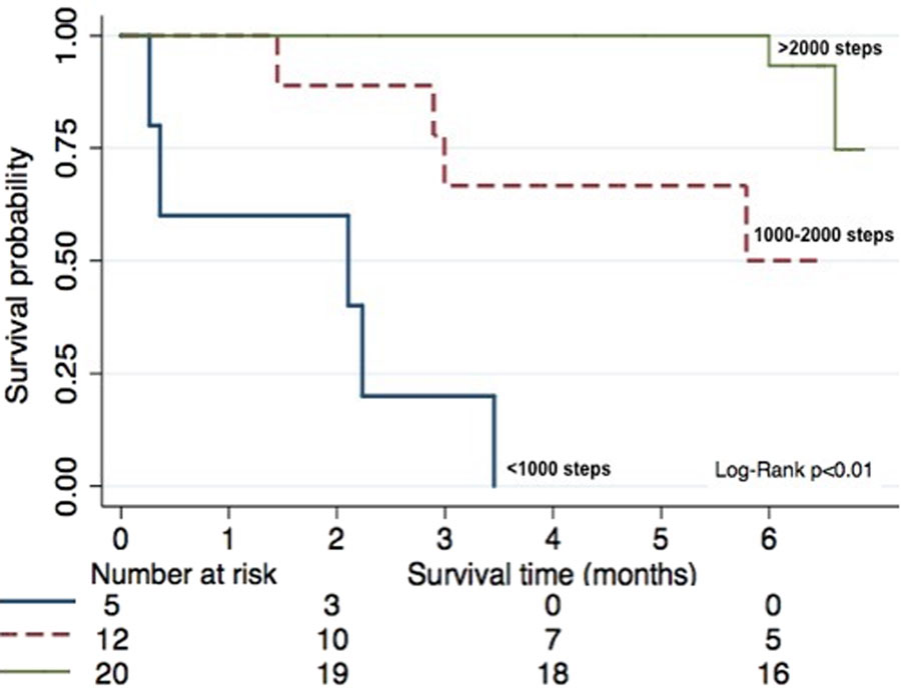
Kaplan–Meier survival curve by step categories

An increase of 1000 steps per day, on average, was associated with significantly lower odds of hospitalizations (OR: 0.21, 95% CI 0.56, 0.79), reduced grade 3 or 4 adverse events (OR: 0.34, 95% CI 0.13, 0.94), and increased survival (HR: 0.48, 95% CI 0.28, 0.83) (Table 2) We also performed regressions for each of the outcomes with steps and either KPS or ECOG included as predictors (data not shown). The results suggest collinearity of predictors where steps were stronger predictors of toxicity and hospitalizations while KPS was a stronger predictor of survival. However, no consistent pattern emerged from this analysis. Kaplan–Meier survival plots are shown in Fig. 3. A median survival of approximately 2 months was observed for patients who walked less than 1000 steps per day, 5.5 months for patients walking between 1000 and 2000 steps, and the median was not attained in patients walking >2500 steps. Stairs climbed were also associated with reduced odds for the occurrence of toxicities and hospitalizations. Nighttime sleep duration was not associated with the occurrence of adverse events or hospitalization but a statistically significant association was observed with overall survival.

**Table 2:** Odds ratios (OR) and hazard ratios (HR) with 95% confidence intervals (CI) from multivariable regression

	Adverse events	Hospitalization	Overall survival
	OR^a^ (95% CI)	OR (95% CI)	HR^b^ (95% CI)
Steps (per 1000 steps)^a^	0.34 (0.13–0.94)	0.21 (0.56–0.79)	0.48 (0.28–0.83)
Floors (per 10 stairs)^a^	0.77 (0.58–1.0)	0.67 (0.48–0.92)	0.78 (0.63–0.96)
Sleep (per 1 hour)^a^	1.78 (0.89–3.5)	1.93 (0.86–4.23)	1.79 (1.14–2.82)

^a^All analyses adjusted for age and sex

^b^Calculated average over 2-week period

### Wearable monitor activity data are correlated with PROs

PROs were completed at each visit using NIH PROMIS® scales: physical functioning, fatigue, pain, sleep, and emotional well-being. All 37 patients completed baseline PROMIS questionnaires and 31 patients completed the PROMIS questionnaires at end-of-study.

The highest correlations were observed between patient-reported physical functioning and average steps (*r* = 0.57), ECOG-PS (*r* = 0.58), and KPS (*r* = 0.68) (*p* < 0.01 for both correlations) (Table 3). Higher levels of patient-reported fatigue were also significantly correlated with decreased step count (*r* = −0.53), shorter distance walked (*r* = −0.51), and fewer stairs climbed (*r* = −0.58). While patient-reported depression was not significantly associated with step count, it was significantly correlated with stairs climbed (*r* = 0.50), ECOG-PS (*r* = 0.58), and KPS (*r* = −0.67). There were no significant correlations between reported sleep and objectively measured sleep. Lower reported quality of sleep was only significantly associated with poor ECOG-PS.

**Table 3:** Correlation coefficients between Fitbit Charge HR® activity metrics, performance status, and NIH PROMIS® *t*-scores

	ECOG-PS	Karnofsky PS	NIH PROMIS® scales^a^				
			Physical functioning	Pain	Fatigue	Sleep	Depression
Steps	−0.63*	0.69*	0.57*	−0.46*	−0.53*	−0.19	−0.36
Distance	−0.61*	0.66*	0.53	−0.49*	−0.51*	−0.14	−0.25
Stairs	−0.57	0.59*	0.43	−0.40*	−0.58*	−0.04	−0.50*
Sleep	0.14	−0.20	0.23	−0.27	−0.24	0.07	0.08
Heart rate	0.52	−0.54	−0.05	0.02	−0.03	0.27	−0.14
ECOG-PS	N/A	−0.85*	−0.58*	0.54*	0.57*	0.33*	0.58*
KPS	0.85	N/A	0.68*	−0.51*	−0.61*	−0.30	−0.67*

^a^Measured at end-of-study visit *p < 0.05

### Reported feasibility and acceptance of the wearable activity monitor

Step counts were the most reliable activity metrics to obtain from patients where all 37 patients had recorded step counts that were properly synced to their device. Heart rate and sleep data were missing in three patients due to syncing errors or removal of device during sleep. Based on individual patient responses in the exit interview, patients described their participation in the study as a positive experience and they considered the device easy to use, unobtrusive, and motivating (Table 4). Common complaints included: difficulty putting on and removing due to the style of the band clasp, not being waterproof, and flashing green light being distracting during sleep. Selected patient responses from the exit interview are provided in Table 4.

**Table 4:** Selected responses from patient exit interview

Themes	Select patient responses^a^
Overall experience	•*“It is a great opportunity to help cancer research.”* •*“I feel motivated when I keep track of my steps and try to beat my steps from the day before. Try to beat my steps each day.”* •*“Positive, good experience. Actually outstanding experience, it was really good for me. Showed me what I can and can’t do, which was more ‘can’. This was a really positive experience*.” •*“It wasn’t very intrusive… it was not a very high maintenance thing. Clock was convenient.”*
Device comfort	•*“It was like a watch.”* •*“I forgot I had it on.”* •*“It was comfortable, except possibly sometimes sleeping…I could feel it on my wrist.”*
Improvements to device	• *“Would be nice if it was waterproof.”* • *“Changing to universal USB, having water-proof.”* • *“Fix the clasp on the band.”* • *“Could it incorporate a sleep-aid?”*
Problems with device	•*“Well the clip is quite difficult. You know the little slip clip that locks it on, but the advantage is once you get it on it stays on. My fingers hurt form neuropathy putting it on and often.”* •*“It was slightly uncomfortable at night.”* •*“Charging. There was one day my activity level was super low because it was charging…”*
Useful to share with doctor	•*“Oh absolutely. Not just the doctor, like I said, the dietician, people who might be able to provide ‘mental support’*.” •*“Yes. I mean, particularly if you’re having sleep trouble, or if they can see how your activity goes up and down in the day and get a sense historically of how the chemotherapy affects you*.” •*“It can explain a lot- Why you’re tired, why you’re not. Open some doors to explain to you and me what’s happening with my body. I didn’t mind wearing it. In fact I’m going to continue wearing it.”* •*“It’s a motivator. You can’t lie about it, either you’re walking or not walking. Keeping a patient accountable. It makes you want to. I think it helps you and makes you understand like on those days you’re not feeling well, I know I was sitting on the couch. Gives you a good measure. You may want to try to walk around the house, or around the living room to get steps in and to help healing process*.”

^a^Exit interviews were conducted during end-of-study visit with 30 patients who agreed to participate in the interview

## Discussion

We examined the use of a consumer-based, wearable activity monitor to assess provider-assessed performance status, PROs, and survival in advanced cancer patients. We found that the wearable activity monitor was feasible for use, especially for the estimation of step counts over a short period of time. The statistically significant correlations observed between objectively measured wearable activity data and validated performance status scales, namely ECOG-PS and KPS, were consistent with our proposed hypothesis that the collection of wearable activity monitor data could supplement clinical evaluation of performance status. Importantly, the activity data suggested a trend for the prediction of clinically relevant adverse events, 30-day morbidity, and 6-month survival. We also observed correlations between wearable activity monitor metrics and PROs including physical functioning, pain, fatigue, and emotional distress.

Our findings provide new information regarding the use of an emerging technology in cancer clinical settings. While other cancer clinical trials have used wearable activity monitors (e.g., pedometers, biosensors, accelerometers) for the objective measurement of physical activity,^14–16^ the use of wearable activity monitors to correlate with functional and clinical outcomes are only beginning to be reported. As a result of the increase in use of wearable activity monitors in clinical settings, there are new oncology trials being developed or currently ongoing that are exploring the use of biosensors with broader applications.^15,19–23^ One such study evaluated the feasibility of the Garmin fitness tracker for predicting hospitalization in cancer patients undergoing concurrent chemotherapy and radiation therapy with curative intent.^23^ Authors reported that patients walked on average 5103 steps per day and an increase in 1000 steps per day was associated with a 38% reduction in risk for hospitalization. These findings were consistent with our own and supported the conclusion that objective activity monitoring in cancer patients is feasible and may be used to predict clinical outcomes such as hospitalization, although this would have to be explored in a larger randomized trial. Clinical trials from other chronic disease areas, such as chronic obstructive pulmonary disease, have also explored the use for wearable activity monitors to track and obtain objective measures of activity and reported their utility in a clinical setting.^24–26^ Thus, while our findings are described within the context of cancer clinical trials, the use of wearable activity monitors for the assessment of physical function and outcome prediction can be applied to broader healthcare settings.

Our findings have several implications for patients, providers, and the public. While our results cannot definitively determine whether activity monitor data can replace ECOG-PS or KPS assessments, they do support their use as a supplement of current functionality tools. Not only were step counts and other activity metrics correlated with performance status, but they also provided a more detailed and continuous account of the patients’ activity levels with the additional benefit of being recorded in the patients’ free-living environments. Their use could also minimize recall biases while removing burden associated with completing multiple surveys and questionnaires in clinic.^4,26,27^ Of additional importance is the possibility of combining objective activity monitoring data with electronically recorded PROs for the purpose of monitoring treatment tolerability and response in future clinical trials. Furthermore, continuous activity monitoring during treatment could help predict and monitor treatment complications and allow for timely and appropriate intervention.^23^

Although limited by its small size and short duration, our trial included participants of varying ECOG-PS and KPS ratings allowing for activity estimates in each of the performance status categories. The majority of our participants were diagnosed with advanced pancreatic cancer disease. Thus, our patients tended to be sicker with poorer prognosis than what we may expect in other cancer groups. This also may explain the lower average PRO scores compared to the standard cancer population and a higher number of adverse events, hospitalizations, and deaths observed during the short study period. While this may limit the generalizability of our findings to healthier and earlier-stage cancer patients and survivors, our findings address a current research gap regarding the use of wearable activity monitors in sick and advanced cancer population. Our population was relatively homogeneous in terms of overall health and treatment schedules adding confidence to our findings and allowing us to show feasibility in a more advanced cancer population. Future studies should enroll more patients from multiple disease sites and stages to further generalize our findings to a broader cancer population. Finally, while device validation will always be a challenge inherent to the use of consumer-based wearable activity monitors in research, the Fitbit Charge HR® has been validated for clinical use, and the accuracy of all wearable activity monitors will continue to improve over time.^28–33^

In conclusion, our study demonstrated the feasibility for use of a wrist-worn, consumer-based activity monitor in advanced cancer patients to objectively measure physical activity with the additional purpose to supplement the measurement of clinical outcomes and PROs. Given the rapid growth in the use of consumer-based fitness monitors worldwide, these findings could also have a larger public health impact with regard to prevention, control, and survivorship programs. For instance, findings from future studies can lead to the development of individualized treatment and exercise plans that may ultimately result in increased treatment tolerability and improved survival outcomes. There is opportunity to enhance patient engagement and communication between patients and providers, as well as motivate patients to monitor and improve their daily activity. Next steps will include studying the longitudinal use of consumer-based wearable activity monitors for clinical outcome measurement and prediction in a randomized, controlled setting across multiple cancer groups.

## Methods

### Study population

Eligible patients were 18 years or older, diagnosed with an advanced solid malignancy (Stage 4 or unresectable advanced Stage 3 cancer) with measurable disease and were treated at a single institution. Patients were required to be ambulatory, but permitted to use walking aids (e.g., cane, crutch) and needed access to a smartphone (personal ownership, family member, friend, or hospital iPad operated by study staff). Patients were excluded if they had a history of allergic reactions to surgical steel or elastomer/rubber or if they had pacemakers and other implantable devices as a safety precaution.

### Study design

We conducted a prospective, single-center, single-cohort trial evaluating the utility of the Fitbit Charge HR® to measure daily activity in advanced cancer patients. The FitBit Charge HR® was selected as it was one of the most popular and inexpensive consumer-based devices during the time the study was designed and is compatible with most smartphones including the Apple iPhone, Samsung Galaxy, Androids and other tablets. It is also water-resistant and has a charge capacity that lasts between 3 and 5 days. Patients were seen in clinic for three consecutive clinic visits (baseline, mid-study, end-of-study), which were no longer than 4 weeks apart (Supplementary Table 2). The trial did not interfere with the patients’ treatment schedules or participation in other trials. Mid-study and end-of-study visits were associated with a 7-day activity period preceding each clinic visit with a total duration of Fitbit activity time of 14 days (2 weeks). Recruitment duration was 1 year and patients were followed for survival for 6 months. The study was completed in August 2017.

### Assessments

All assessments were completed after receiving Research Ethics Board approval and investigators obtained consent from each participant. Consenting patients were provided with a Fitbit Charge HR® and instructed to wear it continuously for the duration of their study period, removing only during showering/bathing/swimming.^16,17^ At each visit, patients completed the NIH PROMIS® questionnaires, and oncologists and nurses independently rated the participants’ ECOG-PS and KPS. The attending oncologist performed a physical exam, collected medical and treatment history, and assessed for AEs.

Fitbit Charge HR® activity outcomes were transmitted through cloud technology to the patient’s mobile device in real-time. We used Fitabase, an online research platform used to manage the multiple Fitbit accounts, to export activity data.^18^

### Study objectives and outcome measures

The primary objective was to measure the association between performance status as assessed using ECOG-PS by the oncologist and average daily step counts as measured using a wearable activity monitor over a 2-week period. We also evaluated the association between Karnofsky performance status and daily step counts as it is equally used in the oncology setting. Secondary objectives included measuring the association between (1) step count and the occurrence of clinical outcomes (survival, adverse events, and hospitalization) and (2) between step count and NIH PROMIS® *t*-scores for pain, sleep, physical functioning, fatigue, and depression. We performed exploratory analyses of the associations between additional activity metrics (stairs climbed, sleep, heart rate) and clinical outcomes of interest.

### Performance status

ECOG-PS (range 0–4) and KPS (range 0–100) were rated at each clinic visit independently by the attending oncologist and nurse. Increasing ECOG-PS on the numerical scale is indicative of worsening performance while increasing KPS is associated with improved performance. A description of ECOG-PS and KPS ratings and their conversion is included in Supplementary Table 1.

### Clinical outcomes

Survival status was assessed at 6 months from end-of-study visit and the occurrence of hospitalizations and grade 3/4 adverse events (CTCAE v 4.03) were measured within 30 days. We also recorded any adverse events related to the wearable activity monitor during the study period. Clinical outcomes were analyzed as binary variables.

### Patient-reported outcomes

NIH PROMIS® short-form questionnaires for pain, fatigue, physical functioning, sleep quality, and depression were administered at baseline, mid-study, and end-of-study.^12^ Each domain is associated with an overall score, which can subsequently be converted to a *t*-score using the NIH PROMIS® toolbox. The PROMIS *t*-scores are normalized and calibrated against a US cancer population with a mean of 50, and standard deviation of 10, making it possible to compare with our own results.^12,13^ Standardized *t*-scores for each domain were analyzed as continuous variables.

### Other variables of interest

Patient demographics, medical history, and comorbidities were collected during the baseline visit. Patient weight, heart rate, and blood pressure were also evaluated at each visit, as measured in the clinic by the practice nurse.

### Statistical methods

The intended size of the study was based on the expected association between provider-assessed ECOG-PS and average daily step counts with an estimated 5000 steps per day and standard deviation of 3300 steps across four ECOG categories.^34^ After accounting for dropout and missing data, a sample size of 30 was required with 80% power and a type I error rate of 0.05.

Activity monitor data were coded as missing if both heart rate data and steps data were equal to 0. Patients were included in the analysis for the primary and secondary outcomes if at least four valid days of activity data were available. Baseline data and PROs were collected and reported for all participating patients.

Spearman rank correlation coefficients were calculated to evaluate relationships between activity metrics, ECOG-PS/KPS ratings, and NIH PROMIS® *t*-scores. Multivariable regression models were used to evaluate the associations between performance status (ECOG-PS, KPS) and wearable activity monitor metrics. Logistic regression models were used for binary outcomes, and linear regression models were used for continuous outcome variables. Models were adjusted for the potential confounding effects of age and sex. To account for the longitudinal measurements, marginal models were employed using generalized estimated equations with an exchangeable correlation structure under the assumption that observations would not vary substantially between visits.

Time-to-event analyses were conducted to evaluate the association between activity measures and 6-month survival and Kaplan–Meier plots were generated. Step counts were categorized into 1000-step increments. Multivariable proportional hazards regression models were also used to evaluate the independent effects of activity data on risk of death. Hazard ratios and their 95% confidence intervals (CI) were computed for each metric, and adjusted for the potential confounding effects of age and sex. All analyses were performed using Stata (Stata Corp, v 14.0).

### Data availability

De-identified data are available upon request from the corresponding author [G.G.] and are not publicly available due to participant privacy.

## Electronic supplementary material

Supplementary Material

Study protocol

## Electronic supplementary material

**Supplementary information** accompanies the paper on the *npj Digital Medicine* website (10.1038/s41746-018-0032-6).
