# Supplementary material for: Wearable activity monitors to assess performance status and predict clinical outcomes in advanced cancer patients
Source: NPJ Digit Med. 2018 Jul 5;1:27. doi: 10.1038/s41746-018-0032-6 (PMC6550281; doi:10.1038/s41746-018-0032-6)
Supplement: Supplementary file 1 — Supplementary Material [file 41746_2018_32_MOESM1_ESM.pdf]

Supplementary material

Supplementary Table 1. Comparison of ECOG and KPS scales

**ECOG Performance Status**[1]	**Karnofsky Performance Status (KPS)**[2]
**0:** Fully active, no restriction in pre-disease performance	**100:** Normal; no evidence of disease
**1:** Restricted in physically strenuous activity but ambulatory and able to carry out light work	**90:** Minor signs or symptoms<br>**80:** Normal activity with effort; some signs or symptoms
**2:** Ambulatory; capable of all self-care but unable to work; up more than 50% of walking hours	**70:** Cares for self; unable to carry on normal activity<br>**60:** Occasional assistance required; capable of most self-care
**3:** Capable of only limited self-care; confined to bed/chair >50% walking hours	**50:** Requires assistance, frequent medical care<br>**40:** Disabled; requires special care/assistance
**4:** Not capable of self-care; totally confined to bed/chair	**30:** Severely disabled; requires special care/assistance<br>**20:** Hospitalization necessary; requires active supportive care<br>**10:** Moribund; progressing rapidly
**5:** Dead	**0:** Dead

1. Karnofsky DA. The clinical evaluation of chemotherapeutic agents in cancer. *Evaluation of chemotherapeutic agents.* 1949
2. Zubrod CG, Schneiderman M, Frei E, Brindley C, Lennard Gold G, et al. Appraisal of methods for the study of chemotherapy of cancer in man: comparative therapeutic trial of nitrogen mustard and triethylene thiophosphoramide. *Journal of Chronic Diseases.* 1960;11(1):7-33.

Supplementary Table 2. Table of events

Procedures	Screening/ Baseline	Mid-Study Visit	End of study	Follow-up (6 months)
Consent	X			
Physical examination (height, weight, heart rate, blood pressure)	X		X	
Demographics (age, sex, occupation al status, education)	X			
Medical History (Comorbidities, concomitant medications)	X			
NIH PROMIS® questionnaires	X	X	X	
Performance Status (ECOG, KPS) assessed by MD and RN	X	X	X	
Frailty (hand grip, walking test, weight loss, physical activity)	X		X	
Feasibility Interview			X	
Clinical Outcomes (Adverse Events, Hospitalization, Survival)			X	X
FitBit Charge HR® daily activity (Steps, stairs, heart rate, sleep)	X	X	X	

Supplementary Table 3. Number of grade 3 or 4 toxicities

Adverse Event[1]	N[2]	%
Ascites	7	19.4
Gastrointestinal hemorrhage	4	11.1
Anemia	3	8.3
Abdominal Pain	2	5.6
Dyspnea	2	5.6
Encephalopathy	2	5.6
Hypernatremia	2	5.6
Sepsis	2	5.6
Weight loss	2	5.6
Aspiration	1	2.8
Biliary Tract infection	1	2.8
Cough	1	2.8
Diarrhea	1	2.8
Hypoxia	1	2.8
Peripheral Sensory Neuropathy	1	2.8
Rash pusutular	1	2.8
Seizure	1	2.8
Skin infection	1	2.8
Thromboembolic event	1	2.8

[1]Excluding elective procedures and laboratory adverse events
[2]Patients could have more than one AE
