# Supplementary material for: Wearable activity monitors to assess performance status and predict clinical outcomes in advanced cancer patients
Source: NPJ Digit Med. 2018 Jul 5;1:27. doi: 10.1038/s41746-018-0032-6 (PMC6550281; doi:10.1038/s41746-018-0032-6)
Supplement: Supplementary file 2 — Study protocol [file 41746_2018_32_MOESM2_ESM.pdf]

IIT2015-10-SHINDE-BIOSENS

The Biosensor Study: Exploratory Study Evaluating the Use of Wearable Biosensors and Patient Reported Outcomes (PROs) to Assess Performance Status and Distress in Patients with Cancer

Principal Investigator: **Arvind Shinde, MD**
Hematology/Oncology and Supportive Care Medicine
Cedars-Sinai Medical Center
8700 Beverly Blvd, Los Angeles, CA 90048

Sub-Investigator(s)	Department/Division
Andrew Hendifar, MD	Hematology/Oncology
Robert Figlin, MD	Hematology/Oncology
Richard Tuli, MD	Radiation Oncology
Christine Walsh, MD	Gynecologic Oncology
Bobbie Rimel, MD	Gynecologic Oncology
Brennan Spiegel, MD	Gastroenterology
**Collaborating Investigators**	**Department/Division**
Steven Piantadosi, MD PhD	Director, Samuel Oschin Comprehensive Cancer Institute
Gillian Gresham	Visiting Graduate Student (Johns Hopkins University) at Cedars-Sinai Medical Center, Samuel Oschin Comprehensive Cancer Institute

Biostatisticians: Andre Rogatko, PhD
Director, Biostatistics and Bioinformatics
Cedars Sinai Medical Center
(310) 423-3316
Andre.Rogatko@cshs.org

Quanlin Li, PhD
Biostatistics and Bioinformatics

IND Number: N/A

Funding Source: Cedars-Sinai Medical Center

Initial version: Protocol Version 8 dated 14 June 2016

CONFIDENTIAL
This material is the property of Cedars-Sinai Medical Center. Do not disclose or use except as authorized in writing by the study sponsor.

Signature Page

The signature below constitutes the approval of this protocol and the attachments, and provides the necessary assurances that this trial will be conducted according to all stipulations of the protocol, including all statements regarding confidentiality, and according to local legal and regulatory requirements and applicable U.S. federal regulations and ICH guidelines.

Principal Investigator (PI) Name: ______________________________

Signature: ______________________________ Date: ____________________

TABLE OF CONTENTS

LIST OF ABBREVIATIONS **1**

STUDY SUMMARY **2**

1.0 BACKGROUND AND RATIONALE **3**

1.1 Background 3

1.2 Rationale 7

2.0 STUDY OBJECTIVES **7**

2.1 Primary Objectives 7

2.2 Secondary Objectives 7

2.3 Endpoints 8

3.0 PATIENT ELIGIBILITY **8**

3.1 Inclusion Criteria 8

3.2 Exclusion Criteria 8

4.0 INTERVENTIONAL PLAN **9**

4.1 Description of Intervention 9

4.2 Duration of Study 10

4.3 Removal of Patients from Protocol 10

5.0 STUDY PROCEDURES **10**

5.1 Screening Procedures 10

5.2 On-Study Procedures. 11

5.3 Time and Events Table 13

5.4 Removal of Subjects from Study 14

6.0 UNANTICIPATED PROBLEMS **14**

6.1 Definitions 14

6.2 Reporting Requirements for Adverse Events.....15

7.0 STATISTICAL CONSIDERATIONS 15

7.1 Study Design/Study Endpoints.....15

7.2 Sample Size and Accrual.....16

7.3 Data Analysis.....16

8.0 STUDY MANAGEMENT.....19

8.1 Conflict of Interest.....19

8.2 Institutional Review Board (IRB) Approval and Consent.....19

8.3 Registration Procedures.....19

8.4 Data and Safety Monitoring.....20

8.4.3 Safety Monitoring.....20

8.5 Amendments to the Protocol.....20

8.6 Record Retention.....21

8.7 Obligations of Investigators.....21

9.0 REFERENCES.....22

10.0 APPENDICES.....23

LIST OF ABBREVIATIONS

ADL	Activities of Daily Living
AE	Adverse Event
BMI	Body Mass Index
BPM	Beats per minute
CGA	Comprehensive Geriatric Assessment
CRF	Case Report Form
CTC	Common Toxicity Criteria
CTCAE	Common Terminology Criteria for Adverse Events
DLW	Doubly Labeled Water
ECOG	Eastern Cooperative Oncology Group
EE	Energy Expenditure
EORTC	European Organisation for Research and Treatment of Cancer
ERC	Ethical Review Committee
H&P	History & Physical Exam
HR	Heart Rate
IRB	Institutional Review Board
IRB	Institutional Review Board
NCI	National Cancer Institute
ORR	Overall Response Rate
OS	Overall Survival
PA	Physical Activity
PS	Performance Status
QMC	Quality Management Core
QOL	Quality of Life
REDCap	Research Electronic Data Capture
SAE	Serious Adverse Event

STUDY SUMMARY

Title	Exploratory study evaluating the use of wearable biosensors and patient reported outcomes (PROs) to assess performance in status and distress in patients with cancer
Protocol Number	IIT2015-10-Shinde-Biosens
Phase	Exploratory
Methodology	Prospective, single-arm, single-center observational study
Study Duration	Nov/2015- Nov/2016
Study Center(s)	Cedars-Sinai Medical Center
Primary Objective	Assess the association between biometric data collected via Fitbit Charge HR®, and PROs collected via NIH PROMIS® short form tools (physical function, pain interference, sleep disturbance, fatigue, and emotional distress), versus validated ECOG-PS and Karnofsky Performance Status scales.
Number of Subjects	30
Diagnosis and Main Inclusion Criteria	Advanced cancer diagnosis (stage 3 or 4)
Study Product(s), Dose, Route, Regimen	FitBit Charge HR ®
Duration of administration	15 days
Statistical Methodology	Correlation statistics to determine association between biosensor data and study outcomes.

1.0 BACKGROUND AND RATIONALE

1.1 Background

Cancer care is complex. Optimizing care decisions in patients with cancer depends on an objective understanding of an individual's clinical condition. Because cancer patients can experience rapid changes in their condition, either as a result of the underlying malignancy or due to morbidity from therapy, real-time data is crucial to making appropriate treatment decisions and assisting in timely delivery of supportive therapies. However, acquisition of real time data from patients utilizing in-person interactions is costly and prohibitive due to limited clinician resources.

Performance Status

Currently, the majority of information upon which oncologists base their treatment decisions is obtained at the time of the patient clinic visit. This data includes the patient's recall of symptoms and physical functioning, in addition to laboratory, imaging and physical exam information. Patient reported data (PRD) can be affected by recall bias. Furthermore, PRD can be influenced by a patient's desire to affect the physician's understanding of their clinical condition. For example, patients may want their oncologist to believe that they are doing well, so that they may be a candidate for further cancer-directed therapies. Patients may de-emphasize physical symptoms and tolerance to therapy in an attempt to convince themselves that they are doing well.

Oncologists rely on the PRD to make their own clinical judgments regarding therapy choices. However, oncologists, themselves, may be influenced by internal factors when making these assessments. The desire to be able to offer further chemotherapy options to patients with advanced disease is strong. Telling patients that they are not candidates for further treatment is difficult. In fact, studies have demonstrated that physicians who have had a longer relationship with a patient, are more likely to over-estimate prognosis, as compared to physicians who do not have a standing relationship with the patient, in part because it is difficult to make objective assessments regarding patients with whom an intense relationship has developed.[1, 2]

Eastern Cooperative Oncology Performance Status (ECOG-PS) is a 0-5 scale used "by doctors and researchers to assess how a patient's disease is progressing, assess how the disease affects the daily living abilities of the patient, and determine appropriate treatment prognosis."[5]. ECOG-PS scores are assessed by asking patients about their recent history of activity when they come to clinic.[3, 4] While initially designed for use as an evaluation tool for patients entering into oncologic research studies managed by the ECOG, higher ECOG PS scores have been demonstrated to correlate with poorer prognosis, and worse outcomes with specific higher-intensity chemotherapy regimens in solid malignancies.[5]

ECOG Performance Status Scale	
**Grade**	**Description**
0	Normal activity. Fully active, able to carry on all pre-disease performance without restriction.
1	Symptoms but ambulatory. Restricted in physically strenuous activity, but ambulatory and able to carry out work of a light or sedentary nature (*e.g.,* light housework, office work).
2	In bed <50% of the time. Ambulatory and capable of all self-care, but unable to carry out any work activities. Up and about more than 50% of waking hours.
3	In bed >50% of the time. Capable of only limited self-care, confined to bed or chair more than 50% of waking hours.
4	100% bedridden. Completely disabled. Cannot carry on any self-care. Totally confined to bed or chair.
5	Dead.

The Karnofsky Performance Status (KPS) is another scale ranging from 0-100%, which measures the ability of cancer patients to perform ordinary tasks. Similar to the ECOG-PS, KPS is assessed by asking patient during a clinic visit about their recent history of functionality.[6] Lower KPS has also been associated with poorer prognosis and outcomes after chemotherapy.[5, 7-9]

Karnofsky Performance Status

100	Normal; no complaints; no evidence of disease
90	Able to carry on normal activity; minor signs or symptoms of disease
80	Normal activity with effort; some signs or symptoms of disease
70	Cares for self; unable to carry on normal activity or to do active work
60	Requires occasional assistance but is able to care for most personal needs
50	Requires considerable assistance and frequent medical care
40	Disabled; requires special care and assistance
30	Severely disabled; hospitalisation is indicated, although death not imminent
20	Very sick; hospitalisation necessary; active support treatment is necessary
10	Moribund; fatal processes
0	Dead

Both ECOG and KPS, which are equally used, are validated scales that are considered the gold standard for the assessment of performance status in cancer patients. ECOG is a 6-item scale

ranging from 0 (normal activity) to 5 (death) and KPS is an 11-point scale with scores ranging from 100 (normal activity) to 0 (death). The reliability and validity of both scales is well-established with their conversion also being validated in the cancer population [5, 14,15] (Table 1). Patient functional status has repeatedly been shown to be an indicator of comorbidity; a predictor of benefit and toxicity of treatments; and an independent prognostic factor. Evaluation of patient function is commonly used to make therapy choices and decide on the type and intensity of a particular cancer treatment. It is also often used to determine patient eligibility in cancer clinical trials. Despite their routine use in oncology, the ECOG and KPS performance scales may provide an over-simplistic and subjective assessment of the patient's activity level. Inaccurate assessments of performance status can result in costly and possibly harmful administration of chemotherapy. Given the inherent subjectivity associated with ECOG and KPS, perhaps an objective measure of a patient's activity level may provide better insight into a patient's prognosis and ability to tolerate chemotherapy.

Supportive Care

Supportive care services in oncology can be delivered before, during or after cancer-directed therapies are employed. They include management of symptoms (i.e. pain, insomnia), medical management of cancer and therapy-related complications (i.e. nausea, dehydration, diarrhea, infection), as well as psychosocial and spiritual support (i.e. depression, anxiety, existential pain). Models of comprehensive cancer care such as the Oncology Care Model as proposed by the Center for Medicare and Medicaid Innovation, require standardized and periodic screening of cancer patients for distress.[10] However, identifying which patients could benefit from each of the services can be resource intensive and challenging. Regular clinician assessments of all cancer patients, as well as paper and electronic survey tools for assessing distress can be helpful to identify patients in need of supportive care services, however they have limitations. Specifically, clinician resources are finite, surveys pose additional clerical burdens on patients, and both forms of assessment evaluate for supportive care needs at a single point in time, while in reality those needs can develop at any point along the disease/treatment trajectory. Perhaps objective real-time monitoring of physiological changes in patients with cancer utilizing their own baseline parameters, could identify patients at high risk for distress, and thereby allow for targeting of finite supportive care resources to the at-risk individuals.

Geriatric Assessments

The National Comprehensive Cancer Network (NCCN) and International Society of Geriatric Oncology have recommended performing a comprehensive geriatric assessment (CGA) as part of routine care in the elderly cancer population. The CGA includes an assessment of frailty which as defined as a phenotype of clinical manifestations: shrinking (weight loss); weakness; poor endurance; slowness; and low physical activity[11, 12]. Frailty has been shown to have important prognostic effects on patients, and is predictive of morbidity, mortality and potential treatment side-effects[11]. Measurement of the clinical manifestations of frailty in the oncology clinic may therefore improve prognostication and treatment selection based on patient's overall well-being as determined by measures of frailty. Unfortunately, similar to distress screening, finite clinical resources can make longitudinal assessments of frailty challenging in practice. Perhaps real-time correlates of frailty can be developed and non-intrusively measured, and thereby minimize the need for resource intensive assessments when treating elderly patients. Table 1 describes the characteristics of frailty that are used to measure the presence of the phenotype.[11]

Table 1. Operationalizing a Phenotype of Frailty

A. *Characteristics of Frailty*	B. *Cardiovascular Health Study Measure**
Shrinking: Weight loss (unintentional) Sarcopenia (loss of muscle mass)	Baseline: >10 lbs lost unintentionally in prior year
Weakness	Grip strength: lowest 20% (by gender, body mass index)
Poor endurance; Exhaustion	"Exhaustion" (self-report)
Slowness	Walking time/15 feet: slowest 20% (by gender, height)
Low activity	Kcals/week: lowest 20% males: <383 Kcals/week females: <270 Kcals/week
	C. *Presence of Frailty*
	Positive for frailty phenotype: ≥3 criteria present
	Intermediate or prefrail: 1 or 2 criteria present

Patient reported outcomes

The integration of patient-reported outcomes (PROs) into cancer care is equally important, as more emphasis is placed on PROs such as symptoms, quality of life, and functional status [15]. Measuring patient values and preferences with regards to their cancer care will improve our understanding of the patient's treatment experience and help tailor their care to their specific needs. PROMIS is such a tool that captures a range of patient-reported outcomes as listed in Appendix A. It includes Likert-type questions that ask patients about their levels of fatigue, physical activity and daily activities. A patient's baseline activity levels may impact PROs such as energy level, pain, distress and ability of self-care, where objective measures of activity may correlate with such PROs and allow for more accurate assessment of the patient's overall treatment experience.

Biometric Sensors – Activity Trackers

The utilization of wearable biometric sensors may allow an inexpensive method of acquiring clinical data. This information could be used to improve clinical decision-making and provide actionable information regarding acute changes in a patient's clinical condition. Accurate and objective information would be expected to improve the quality and appropriateness of treatment decisions, thereby minimizing potentially harmful and costly inappropriate treatment choices. Real-time information regarding clinical deterioration could allow for timely medical interventions that could improve clinical outcomes and reduce cost by addressing a patient's medical needs before the condition presents in a more advanced state.

Currently, off-the-shelf technology is being used commercially to track fitness. These wrist-worn devices can provide real-time data relating to movement, altitude, heart rate, body composition, temperature, and sleep quality. These devices could be worn by patients with cancer to provide valuable data which then could be utilized to inform patient-centric clinical decisions and ultimately improve clinical outcomes.

This protocol describes early feasibility testing of a proposed strategy to collect activity tracker data in a cohort of patients with cancer and correlate the data with validated metrics for performance status. Furthermore, collected data will be related to validated symptom/distress screening tools to assess for correlation between physiologic changes and distress. Researchers at CSMC will provide to patients with advanced cancer, commercially-available wrist activity trackers to collect physiological data, including steps walked, stairs climbed, total activity, calories burned, sleep quality, and heart rate. Validated electronic tools will be employed as distress

screens/quality of life assessments. Clinicians will be requested to include performance status evaluations as part of the standard clinic visit.

1.2 Rationale

The results of this exploratory study will optimize the development of a larger study to evaluate the use of wearable bio-sensors to obtain performance status correlates and their relationship to meaningful outcomes such as toxicity from chemotherapy and survival, as well as identify patients with physiologic/psychosocial distress for targeted supportive care interventions. Furthermore, the qualitative experience of patients captured during this study, including their knowledge, attitudes, beliefs, and suggestions regarding PROs and biosensor tracing, will be used to design future studies to improve care in patients with cancer. This study will be the first of many steps to inform the development of a future electronic platform that will integrate physiologic data from wearable biosensors and provide real-time, objective, and actionable information to oncologists to inform patient-centric clinical decisions and ultimately improve outcomes.

As technology advances, biosensors will play an increasing role in the care of patients with cancer. Accurate and minimally intrusive real-time assessments will assist in providing appropriate and patient-centric therapies to cancer patients. This study is a preliminary evaluation of utilizing real-time physiologic data to assess for functional status. It will also provide greater understanding as to the utility of passively assessing real-time physiologic data to screen for distress, with the goal of ultimately developing a platform to screen for physical and psychosocial distress. Biosensor data could be further used to ascertain outcomes such as measures of frailty, should an association be observed.

2.0 STUDY OBJECTIVES

2.1 Primary Objectives

2.1.1 Assess the association between biometric data collected via Fitbit Charge HR®, and PROs collected via NIH PROMIS® short form tools (physical function, pain interference, sleep disturbance, fatigue, and emotional distress), versus validated ECOG-PS and Karnofsky Performance Status scales.

2.2 Secondary Objectives

2.2.1 Assess the association between biometric data and indicators of distress as measured on validated NIH PROMIS® short form tools (physical function, pain interference, sleep disturbance, fatigue, and emotional distress).

2.2.2 Evaluate frailty within the study population and how measures of frailty correlate with biosensor data

2.2.3 Assess the feasibility of collecting biometric data and PROMIS® results in patients with solid malignancies.

2.2.4 Assess for relationship between biometric data collected via Fitbit Charge HR®, and PROs collected via NIH PROMIS® short form tools (physical function, pain

interference, sleep disturbance, fatigue, and emotional distress), versus clinically meaningful outcomes of chemotherapy toxicity and survival.

2.3 Endpoints

2.3.1 Biometric data from Fitbit Charge HR and PROMIS® scores will be compared to reference standards, ECOG PS and KPS to answer primary objective 2.1.1.

2.3.2 Correlation statistics between biometric data and PROMIS tools at 8 days and 15 days will be measured to answer secondary objective 2.2.1.

2.3.3 Frailty is present if ≥3 characteristics of the following are present: unintentional weight loss, handgrip strength at lowest 20%, exhaustion (self-report) from PRO; walking time in 15 feet (slowest 20%); and low activity (lowest 20% Kcals/week).[12] Association between measures of frailty and biosensor data will be measured to answer secondary objective 2.2.2. See Appendix D for description of each characteristic.

2.3.4 The secondary objective 2.2.3 feasibility will be assessed as follows: Feasibility of collecting biosensor data will be measured using the duration of time that the Fitbit Charge HR is worn and recording data during the study. Recorded data from the device for 4 of each 7 days of the study (at least 16 hrs of each day) will support feasibility of utilizing the Fitbit Charge HR to collect data. Feasibility of collecting PROMIS tool responses will be determined by percentage of completed PROMIS tools. PROMIS tool completion rates of >50% will support the feasibility of current method of utilizing PROMIS tools to collect PROs.

2.3.5 Chemotherapy associated toxicity (utilizing CTCAE v4) occurring within 4 weeks of chemotherapy will be assessed for patients who are administered chemotherapy during the 15-day study period to answer secondary objective 2.2.4.

2.3.6 Information regarding adverse events occurring within 3 months of the close of the 15-day active observation period of the study, i.e. hospitalizations (including reason for hospitalization), will be collected to answer secondary objective 2.2.4.

2.3.7 Survival data (including date and cause of death) will be collected to answer secondary objective 2.2.4.

3.0 PATIENT ELIGIBILITY

3.1 Inclusion Criteria

3.1.1 Diagnosis of advanced solid malignancy (Stage 3 or 4) with measurable disease, who are being followed by an oncologist
3.1.2 18 years or older
3.1.3 English speaking
3.1.4 Ambulatory (use of walking aids, such as cane and rollator, is acceptable)
3.1.5 Access to a device that has the capability to sync to the Fitbit
3.1.6 Expected to have oncology clinic visits at least once every 4 weeks to line up with their treatment schedule.
3.1.7 Have an understanding, ability, and willingness to fully comply with study procedures and restrictions
3.1.8 Ability to consent

3.2 Exclusion Criteria

3.2.1 Allergy to surgical steel or elastomer/rubber

3.2.2 Using a pacemaker, implantable cardiac defibrillator, neurostimulator, hearing aids, cochlear implants, or other electronic medical equipment

Patient Safety

The sensor uses a Bluetooth Low Energy (BTLE) Transceiver, an accelerometer, and microcontroller; therefore, individuals using pacemakers, implantable cardiac defibrillators, neuro-stimulation devices, cochlear implants and hearing aids, or using other electronic medical equipment will be excluded from the study. The wristbands associated with the activity trackers are made from an elastomer. There is also surgical stainless steel incorporated into the device. For this reason, individuals with allergies to elastomer/rubber or surgical steel will be excluded from the study.

4.0 INTERVENTIONAL PLAN

4.1 Description of Intervention

4.1.1 Activity Tracker

A primary goal of this exploratory study is to evaluate the use of commercially available fitness trackers in oncology patients to assess for performance status. The Fitbit Charge HR (Figure 1) will be utilized in this study.

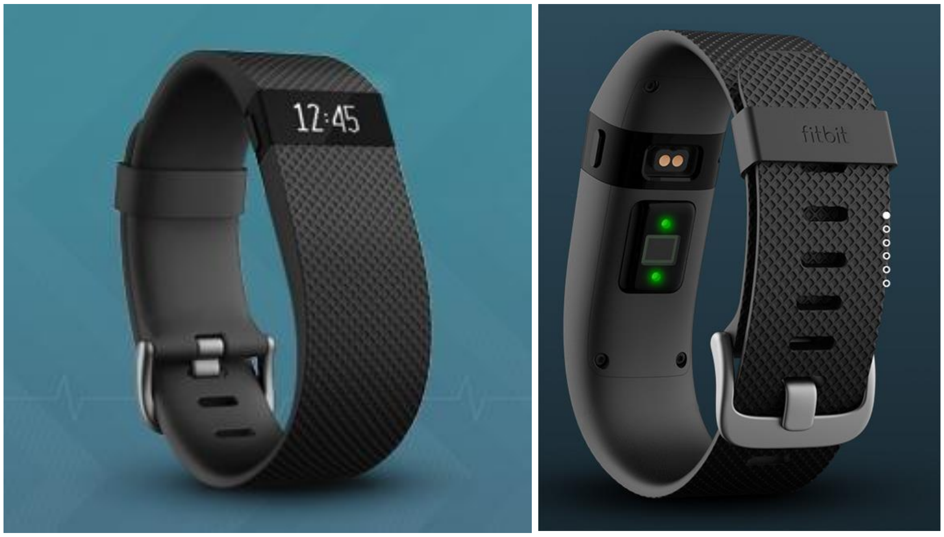

Figure 1: Fitbit Charge HR activity tracker

The Charge HR was chosen due to a combination of factors, including sensor capability, battery life, water resistance, Bluetooth capabilities, and presentation of data. Furthermore, a wrist worn device is being utilized as such devices allow for automated heart rate monitoring. The data collected via the Fitbit Charge HR is analyzed and displayed via Fitbit's proprietary software and is available to authorized individuals via smartphone and web-based applications. Fitbit also allows 3rd parties to develop software and algorithms to analyze the collected data, and allow free sharing of the data as long as permission is received from the device owner. Data from the device is transmitted via Bluetooth to an iOS or Android equipped smartphone. Data from the smartphone is then

communicated over the internet to company servers, where third-party applications with permission can have access to physiologic data. There will be no electronic link between subject physiologic data on the manufacturer servers and the patient identified data in the CSMC electronic medical record. De-identified aliases will be used to enroll the devices on the manufacturer's website. The subject key, which will associate the aliases with patient names, will be kept in a locked cabinet within the PI's office on the CSMC campus. At the completion of the two week study, patients will be surveyed to assess for the experience of wearing the activity trackers and whether any difficulties were noted.

4.1.2 Patient Reported Outcome (PRO) Measures

A primary goal of the study is to examine the relationship between symptoms and quality of life (as measured by PROs), and activity tracker data. To accomplish this goal, subjects will be requested to complete an online NIH PROMIS computerized survey which will assess for domains of quality of life and symptoms. A composite survey of 28 questions will be generated utilizing the REDCap online survey tool with the following PROMIS Short Form Surveys: v1.0 Physical Function – Short Form 20a, v1.0 Pain Interference – Short Form 4a, v1.0 Sleep Disturbance – Short Form 4a, v1.0 Fatigue – Short Form 8a, and v1.0 Emotional Distress – Depression –Short Form 4a (Figure 4). The following domains will be evaluated: global functioning; sleep quality; pain; fatigue; and emotional distress. Subjects will receive a weekly email with a link to complete the survey before the Day 8 and Day 15 clinic visit. Patients who do not complete the survey can complete the survey at their next clinic visit. Changes in survey responses will be observed over time, and correlations with recorded physiologic data obtained from the biometric sensors will be assessed. It is our hope that specific physiologic changes will correlate with the presence of symptoms of distress, and thereby function as a real-time, but passive, assessment for the development of distress. All of the survey data entered will be encrypted and housed on an IRB approved, HIPAA-compliant RedCap server. Any collected data is only accessible by the research team and will be de-identified before being aggregated for research purposes or academic publication. Hard copy surveys will be stored in a locked cabinet within the office of the research coordinators.

4.2 Duration of Study

Subjects will be on study for a 15-day period and may be contacted twice by phone in the six months following the 15 days.

4.3 Removal of Patients from Protocol

Patients will be removed from the study when any of the criteria listed in Section 5.4 apply. Notify the Principal Investigator, and document the reason for study removal and the date the patient was removed in the Case Report Form. The patient should be followed-up per protocol.

5.0 STUDY PROCEDURES

+/- 3 days for clinic visits

5.1 Screening Procedures

It is acceptable for screening procedures to take place on Day 1 of Study.

Assessments performed exclusively to determine eligibility for this study will be done only after obtaining informed consent. Assessments performed for clinical indications (not exclusively to determine study eligibility) may be used for baseline values even if the studies were done before informed consent was obtained.

5.1.1 *Informed Consent – Must occur within 6 weeks of Day 1 of study*

5.1.2 *Medical history and Limited Physical Exam*

Complete medical and surgical history will be collected. This will include the collection of comorbidities, previous cancers, previous treatments and surgical procedures and current physical assessment by body system. Patients will be asked whether they have experienced unintended weight loss over the past year and how much. Information about current disease will also be collected including cancer type and stage. Both KPS and ECOG performance status will be collected.

5.1.3 *Treatment Plan Data Collection*

5.1.4 *Demographics*

Patient demographics will be collected including: age, sex, race, ethnicity, education status, employment status and substance use.

5.1.5 *Review subject eligibility criteria*

5.1.6 *Subject Registration*

5.2 On-Study Procedures

FitBit Charge HR is worn continuously for 15 days and removed before showering or bathing and before any activity that would submerge the FitBit in water.

5.2.1 Visit 1 (Day 1) – Clinic Visit

- Performance Status (ECOG and KPS) – Physician assessed
- Performance Status – Assessed by nurse
- Assessment of baseline frailty: Assessment of gait speed (as measured using timed walk in 15 feet) and hand-grip strength using dynamomometer – study coordinator assessed (Day 1 only)
- Limited physical exam, including height, weight, heart rate, and blood pressure measurements
- Fitbit Charge HR Instructions for charging and syncing on Day 1
- PROMIS tool – subject will complete the self-report either by email or in –person while at the clinic visit

5.2.2 Mid-study visit- Clinic Visit

- Fitbit Charge HR Functionality testing (Check battery life and device syncing from the Fitbit application on hospital computer). Patient attendance not required to do this. Patient may be contacted by telephone if they have not charged or synced recently.
- PROMIS tool – subject will complete the self-report online by email if not in clinic. If patient is seen on a weekly schedule, they may have the option to complete this on paper during treatment or in the clinic.

5.2.3 End of Study visit – Clinic Visit

- Performance Status – Physician assessed
- Performance Status -Assessed by nurse
- Assessment of frailty: Gait speed test (timed walk of 15 feet) and hand-grip strength using dynamomometer – Study coordinator assessed

- Limited physical exam, including height, weight, heart rate, and blood pressure measurements
- Fitbit Charge HR functionality assessment. Patient has the choice to keep Fitbit at end of study. Otherwise, patient can return the Fitbit at this time. Patient must return wall-charging block.
- PROMIS tool – subject will complete the self-report either by email or in–person while at the clinic visit
- End-of-Study Subject-Evaluation of study experience with Fitbit (exit interview) may be audio recorded and can occur at Day 15 or after
- ECOG/KPS – subject will complete the self-report either by email or in–person while at the clinic visit

5.2.3 Follow-up Phase

- 4 Weeks after Study Period – Chart review *(for chemotherapy toxicity evaluation after study completion for patients who received chemotherapy during the 15-Day study period)*
- 12 weeks (3 months) after Study Period – Chart review and/or telephone call to subjects *(collect hospitalization events or date and cause of death).* No need to conduct phone interview if chart review reveals the subject has died.
- 24 weeks (6 months) after Study Period – Chart review and/or telephone call to subjects *(collect hospitalization events or date and cause of death)*. No need to conduct phone interview if chart review reveals the subject has died.

5.3 Time and Events Table

	Screening	Visit 1-Day 1 +/- 3 days for clinic visits	Mid-study visit[5, 8] +/- 3 days for clinic visits	End-of-study visit[8] +/- 3 days for clinic visits	Post Study Follow-up
Informed Consent	X				
Medical History	X				
Limited physical exam[1]		X	X	X	
Treatment Plan Data Collection	X				
Demographics	X				
Eligibility Evaluation	X				
Physician assessment: Performance Status ECOG/KPS		X	X	X	
Nurse assessment: Performance Status ECOG/KPS		X	X	X	
FitBit Instructions		X			
Assess FitBit Functionality			X	X	
FitBit Charge HR Use		Continuous use Days 1-15			
Frailty assessment (including hand grip strength and timed walk in 15 feet)[2]		X		X	
PROMIS		X	X	X	
End-of-Study: ECOG/KPS self-evaluation, study experience evaluation, and FitBit return[6]				X	
Adverse Events[7]					X[3]
Chart review and/or Telephone call for hospitalizations or survival					X[4]

[1]Includes height, weight, heart rate, and blood pressure
[2]See Appendix D for specific protocol and definitions related to assessment of frailty
[3]Chart review for chemotoxicity, if applicable, at 4 weeks +/- 7 days after completion of 15-day study period
[4]At 3 months and 6 months after completion of 15-day study period, +/- 7 days
[5]Patients must have at least 7 days of activity data prior to mid-study visit. Patients should complete the PROMIS questionnaire on day of mid-study visit.
[6]Exit interview can occur at End-of-Study visitor after
[7]Any adverse event related to current protocol (e.g., anticipated Fitbit-related AEs) and any other serious adverse event (defined as grade 3 or higher AE in CTCAE v4.03) treatment or cancer-related to be collected
[8] Visits should be no more than 4 weeks apart. Visit schedule will be based on patient's individual standard-of-care visit schedule, where patients may be seen every week, 2 weeks, 3 weeks or 4 weeks. Patients seen more than a week apart will be reminded by phone or email to wear their Fitbit at least 7 days prior to the scheduled visit at all times and complete the online PROMIS questionnaires.

5.4 Removal of Subjects from Study

Patients can be taken off the study treatment and/or study at any time at their own request, or they may be withdrawn at the discretion of the investigator for safety, behavioral or administrative reasons. The reason(s) for discontinuation will be documented and may include:

5.5.1 Patient voluntarily withdraws (follow-up permitted)

5.5.2 Patient withdraws consent (termination of treatment and follow-up)

5.5.3 Patient is unable to comply with protocol requirements

5.5.4 Treating physician judges continuation on the study would not be in the patient's best interest

5.5.5 Lost to follow-up

6.0 UNANTICIPATED PROBLEMS

6.1 Definitions

6.1.1 Unanticipated Problems

An unanticipated problem involving risk to subjects or others (UP) is defined as any unexpected incident, event, or problem that is related or possibly related to the research and poses greater risk of harm than was previously known to an individual or group of individuals (including research subjects, research staff, or others not directly involved in the research).

Examples of Unanticipated Problems may include:

- Subject complaints
- Other errors in the conduct of the research
- Protocol deviations or violations
- Protocol exceptions (changes made to the research without prior approval in order to eliminate apparent immediate harm to subjects)
- Breach of confidentiality
- Billing problems that pose unanticipated financial risk to subjects
- Any discomfort, pain or other effects associated with the use of the sensor or other research materials will be recorded.

6.1.2 Serious Adverse Events

A "serious" adverse event is defined in regulatory terminology as any untoward medical occurrence that:

6.1.2.1 Results in death.

If death results from (progression of) the disease, the disease should be reported as event (SAE) itself.

6.1.2.2 Is life-threatening.

(the patient was at risk of death at the time of the event; it does not refer to an event that hypothetically might have caused death if it were more severe).

6.1.2.3 Requires in-patient hospitalization or prolongation of existing hospitalization for ≥ 24 hours.

6.1.2.4 Results in persistent or significant disability or incapacity.

6.1.2.5 Is a congenital anomaly/birth defect

6.1.2.6 Is an important medical event

Any event that does not meet the above criteria, but that in the judgment of the investigator jeopardizes the patient, may be considered for reporting as a serious adverse event. The event may require medical or surgical intervention to prevent one of the outcomes listed in the definition of "Serious Adverse Event".
For example: allergic bronchospasm requiring intensive treatment in an emergency room or at home; convulsions that may not result in hospitalization; development of drug abuse or drug dependency.

6.2 Reporting Requirements for Adverse Events

We expect this study to be low risk to patients and do not anticipate any serious adverse events related to the study intervention. However, in the case that there are unanticipated serious events, they will be reported as per institutional standards:

6.2.1 Expedited Reporting

- The Principal Investigator must be notified within 24 hours of learning of any serious adverse events, regardless of attribution, occurring during the study.
- The IRB must be notified of "any unanticipated problems involving risk to subjects or others" or any serious adverse event determined by the PI to be at least possibly related to the research study within 10 business days of the investigator's awareness of the event.

7.0 STATISTICAL CONSIDERATIONS

7.1 Study Design/Study Endpoints

This is an observational study that will follow patients with stage 3-4 cancer for a period of 15 days. Subjects will be provided with a Fitbit Charge HR activity tracker. They will be requested to wear the sensors at all-times, except for while bathing or performing other activities where the device can become submerged in water. Subjects will be asked to wear the device for 15 days. We will recruit as many patients as we can from each ECOG category.

Subjects will be evaluated at three times over the study period (Baseline visit (day 1), Mid-study visit (at next clinic visit), and end-of-study visit) while visiting their oncologists as part of the standard medical care. Initial evaluation will include collecting data about patient's diagnosis, height and weight, and current treatment plan over next 2 weeks (chemotherapy, radiation therapy, no therapy). Oncologists will be requested to record ECOG-PS and KPS during clinic visits that occur in the two-week study period. Furthermore, a research/practice nurse will meet patients during their oncology appointment and provide their own assessment of ECOG-PS and KPS during pre-specified points during the study, specifically on day 1, day 8, and day 15 of the study.

During the active study period, subjects will be requested to complete weekly symptom and quality of life assessments, utilizing a modified PROMIS questionnaire (Figure 4). They will

receive a weekly email with a link to the questionnaire on days 1, day 8, and day 15. In addition to the PROMIS tool, subjects will receive a 2 question survey as part of the day 15 assessment describing categories corresponding to the ECOG and KPS scale, and will be asked which category they believe best represents their functional status over the past week. Patients, who are unable to complete the web-based questionnaire, will be given the opportunity to complete a paper form of the questionnaire during clinic visits at the same time-points (+/- 3 days).

After completion of the active data collection portion of the study, patients will have the option to keep the activity tracker or return to study coordinator. Should the activity tracker be returned, it will be disinfected utilizing and alcohol-based solution. All data from the study patient will be downloaded from the device, and deleted from the device. Once sanitized, the device will be reset and then provided to another subject for activity tracking purposes.

After completion of the active monitoring of patients, follow-up data will be collected regarding toxicity to chemotherapy, hospitalizations, and survival. Specifically, follow-up data regarding chemotherapy-associated toxicity within 4 weeks of the administration of chemotherapy will be collected via chart review. Information regarding hospitalizations occurring within 12 weeks of completion of 15-day active observation, including dates and cause of hospitalization, will be documented via chart review and phone calls. Survival data, including date of death, and cause of death will be collected via phone calls and social security death index for up to 3 years. Patients who remain alive after 3 years will have a censored observation.

7.2 Sample Size and Accrual

7.2.1 Sample Size Consideration

Thirty subjects will be included in this prospective pilot intervention.

Due to the lack of prior data on the association between ECOG PS scores and the activity metrics measured from wearable biosensors the power was determined using a linear regression for steps taken per day and the ECOG scale. The average and standard deviation of 5000 and 3300 per day will be used as an estimate for the step taken for ECOG Grade 0/normal and 0 will be used for ECOG Grade 4/completely disabled.[13] A sample size of 23 patients would be able to detect a change in slope from 0 to 1250 using a linear regression model and assuming 80% power and a type I error rate of 0.05. To accommodate dropout and missing data, we will enroll a total of 30 subjects in the study.

7.2.2 Patient Recruitment

Patients with a solid tumor diagnosis (Stage 3 or 4) and either receiving, or with a plan to receive chemotherapy or radiation therapy in the next 1 month, will be approached during their outpatient visit to their medical oncologist by one of the study co-investigators. Patients who are already enrolled in a clinical trial will remain eligible for this study. The potential subject will be evaluated to see if they are eligible for the study and if so, the potential subject is presented with an option of enrolling. We will enroll as many patients as we can from each ECOG-PS category. We will use the ECOG rated by the MD from day 1 (+/-7 days) for determination of ECOG category.

Patients will have time and the opportunity to review the study materials and consent forms, and will be provided contact information for any questions prior to the baseline visit should they consent to participation.

7.3 Data Analysis

7.3.1 Quantitative Data Collection

During the 15-day data collection period, study subjects will be requested to allow the activity tracker to sync with the device application on their phone. Data collected will be available to study investigators via the Fitbit

web-based GUI (Figure 2). If there is a problem with syncing the Fitbit Charge HR with a patient's smartphone the data can be accessed during clinic visits and synced to a researcher's tablet or smartphone. For the purpose of this pilot study, pre-formatted data as presented by the manufacturer will be utilized for the data analysis. This will include heart rate, steps taken, floors climbed, total activity, active minutes, duration of sleep, restless periods during sleep, awakenings during sleep, and sleep latency. For future studies, we expect to incorporate raw data from the activity trackers into correlation analysis. An export (.csv or .xls file) of these data is available through the fitbit device application and through Fitabase.

Fitabase is a research platform that will allow us to manage multiple Fitbit accounts through the Fitbit dashboard. It is used used to aggregate, analyze, and export data from the fitbit devices. The same data from Fitbit is collected in Fitabase, including the battery life, syncing times and activity data (step count; heart rate; sleep duration and efficiency; calories; intensity). Fitabase uses Secure Sockets Layer for administration of their site and encrypts all passwords. Data is collected anonymously where only information on steps walked, calories burned, sleep duration, and other physical activity classifications. It does not collect IP addresses from the synced devices. A unique study ID (numbers 001-030) coded in Fitabase will be used to manage the Fitbit activity data by participant and linkage will be achieved externally by merging the REDCap data export with the activity data, by study ID. The use of Fitabase for management of Fitbit data will not affect the integrity of the data, nor introduce any additional risk to patient confidentiality and security.

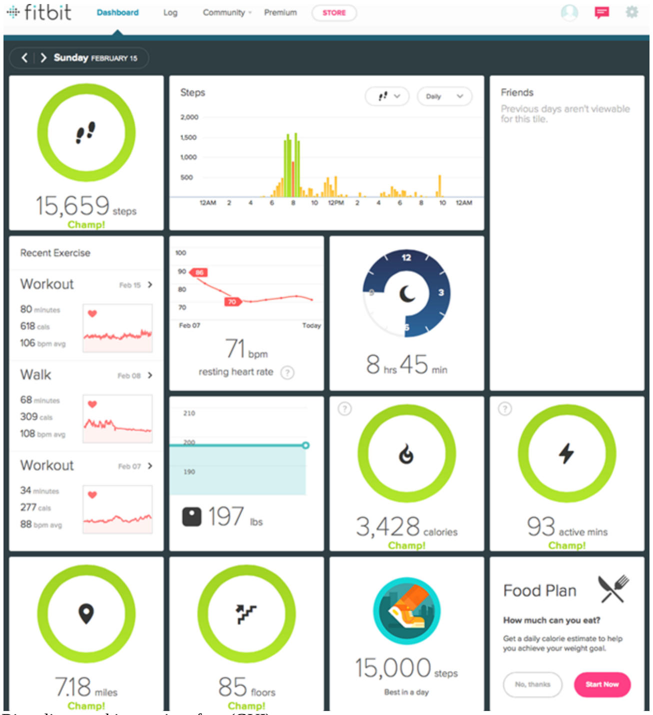

Figure 2a: FitBit online graphic user interface (GUI)

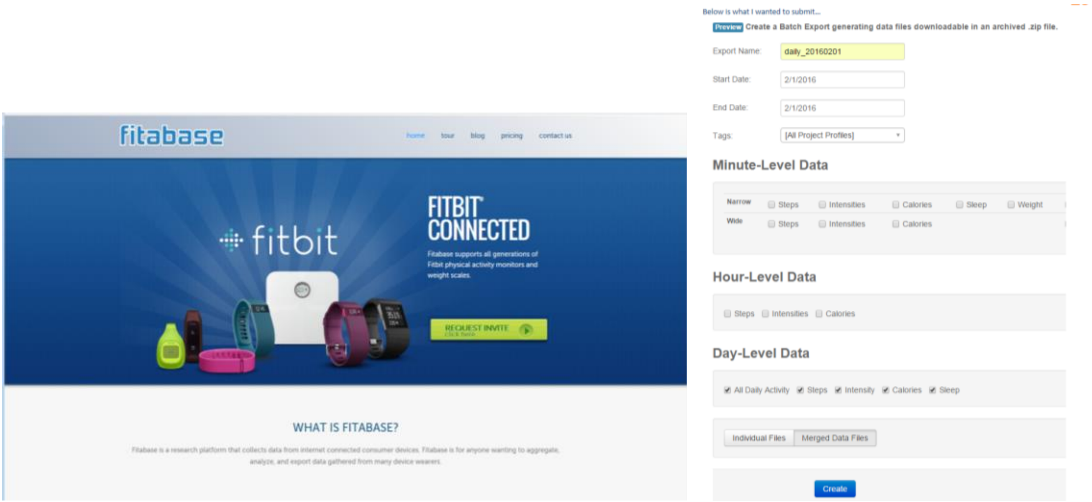

Figure 2b. Fitabase online user interface

On days 1, 8 and 15, treating oncologists will be requested to determine ECOG and KPS. A research nurse will also provide independent assessment of ECOG and KPS on days 1, 8, and 15. The research nurse providing an independent assessment of performance status will be blinded to the data collected from the Fitbit and PROMIS tools.

At the completion of the study, patients will be given a survey to assess their experience with wearing the activity tracker (Figure 6). Specific questions will assess the following issues relating to the experience:

- Charging
- Uploading data
- Comfort
- Issues of concern
- Frequency of viewing data as displayed on smartphone, and how this may have influenced activity

Data pertaining to graded toxicity to chemotherapy, hospitalizations, and survival will be collected during the follow-up period.

7.3.2 Data Analysis Plan

In this study, we hope to examine whether there is an association between biometric data (steps taken, total activity, stairs climbed and duration of sleep) measured by FitBit Charge HR activity tracker, PROs as evaluated by PROMIS tools and performance status as determined by validated ECOG Performance Status scores. Our primary hypothesis is that physiologic metrics collected by wearable biosensors (FitBit Charge HR activity tracker) are associated with ECOG Performance Status score.

The descriptive phase of analysis will include an assessment of the distributions and correlations of variables of interest including: steps taken, total activity, stairs climbed and duration of sleep measured by FitBit Charge HR activity tracker. The main analysis examining the association of these physiologic metrics with the validated ECOG scores will be done using a regression model. The distribution of the physiologic metrics will be examined, and any transformations of the outcomes will be performed through an analysis of residuals. Non-normal distribution will be corrected using a transformation (e.g. log, Box-Cox). In addition to the distribution, other model fit characteristics such as influence diagnostics, collinearity and potential interactions among the activity measures will be examined in an exploratory analysis of the data before deciding upon the model.

Standard variable selection methods such as stepwise and backward procedures will be used to determine the best subset of covariates to include in the model.

8.0 STUDY MANAGEMENT

8.1 Conflict of Interest

Any reportable conflict of interest will be disclosed to the local IRB and will be outlined in the Informed Consent Form.

8.2 Institutional Review Board (IRB) Approval and Consent

It is expected that the IRB will have the proper representation and function in accordance with federally mandated regulations. The IRB should approve the consent form and protocol.

In obtaining and documenting informed consent, the investigator should comply with the applicable regulatory requirement(s), and should adhere to Good Clinical Practice (GCP) and to ethical principles that have their origin in the Declaration of Helsinki.

Before recruitment and enrollment onto this study, the patient will be given a full explanation of the study and will be given the opportunity to review the consent form. Each consent form must include all the relevant elements currently required by the FDA Regulations and local or state regulations. Once this essential information has been provided to the patient and the investigator is assured that the patient understands the implications of participating in the study, the patient will be asked to give consent to participate in the study by signing an IRB-approved consent form.

Prior to a patient's participation in the trial, the written informed consent form should be signed and personally dated by the patient and by the person who conducted the informed consent discussion.

8.3 Registration Procedures

All subjects that sign informed consent will be assigned a subject number sequentially by their date of consent. Those subjects that do not pass the screening phase will be listed as screen failures on the master list of consented subjects. Eligible subjects, as determined by screening procedures and verified by a treating investigator, will be registered on study at Cedars Sinai Medical Center by the Study Coordinator.

Issues that would cause treatment delays after registration should be discussed with the Principal Investigator (PI). If a patient does not receive protocol therapy following registration, the patient's registration on the study may be canceled. The Study Coordinator should be notified of cancellations as soon as possible.

The study team will track all subjects who sign consent on a subject screening/enrollment log using a unique screening ID (S01, S02, etc.). Subjects found to be ineligible will be recorded as screen failures. Subjects found to be eligible will be registered.

A) <u>Eligibility Verification</u>

Prior to registration, all subjects must undergo eligibility verification by the SOCCI Clinical Research Office (CRO). The following documents will be completed and provided for review:

- Registration form (or equivalent)
- Copy of relevant source documents
- Eligibility checklist (signed by investigator)
- Signed patient consent form and Subject's Bill of Rights

- HIPAA authorization form

B) <u>Registration</u>

After eligibility is verified, registration is completed as follows:

- Assign a patient study number
- Enter the patient in OnCore
- Notify the treating physicians that a subject has gone on study

Oversight by the principal investigator is required throughout the entire registration process

8.4 Data and Safety Monitoring

8.4.1 Data Security and Privacy

Data collected by the Fitbit Charge HR will be de-indentified. An alias will be associated with each device, and therefore data analysis provided by the Fitbit website will not be associated with identifying information. There will be no physical or virtual link between the physiologic data collected by the Fitbit Charge HR, and the patient's electronic medical record.

Survey data collected via the REDCap survey tool will not be linked to the patient's electronic medical record. Reports of survey responses will be stored on password protected computers.

The key, which associates the alias with PHI data will be stored on a Cedars-Sinai provided, password protected computer.

8.4.2 Data and Quality Assurance

The Samuel Oschin Comprehensive Cancer Institute Clinical Research Office (CRO) Quality Management Core (QMC) will conduct routine monitoring for protocol adherence and data quality for investigator-initiated cancer trials. QMC will review study records and directly compare them with source documents, discuss the conduct of the study with the investigator, and verify that study is conducted in accordance with applicable regulations.

The investigator must notify QMC promptly of any inspections scheduled by regulatory authorities (e.g. FDA), and promptly forward copies of inspection reports to QMC.

8.4.3 Safety Monitoring

As a single-site study, all safety information will be reported directly to the PI upon occurrence. The PI will maintain continuous safety monitoring for the duration of the study. If the PI becomes aware of any new safety information that may place subjects at increased risk than what was previously known, enrollment will be held until the PI determines whether a modification to the study is necessary and/or the informed consent documents are updated accordingly.

8.5 Amendments to the Protocol

Should amendments to the protocol be required, the amendments will be originated and documented by the Principal Investigator. It should also be noted that when an amendment to the protocol substantially alters the study design or the potential risk to the patient, a revised consent form might be required.

The written amendment, and if required the amended consent form, must be sent to the IRB for approval prior to implementation.

8.6 Record Retention

Study documentation includes all Case Report Forms, data correction forms or queries, source documents, monitoring logs/letters, and regulatory documents (e.g., protocol and amendments, IRB correspondence and approval, signed patient consent forms).

Source documents include all recordings of observations or notations of clinical activities and all reports and records necessary for the evaluation and reconstruction of the clinical research study.

Government agency regulations and directives require that the study investigator must retain all study documentation pertaining to the conduct of a clinical trial. Study documents should be kept on file until three years after the completion and final study report of this investigational study or as required by institutional guidelines.

8.7 Obligations of Investigators

The Principal Investigator is responsible for the conduct of the clinical trial at the site in accordance with Title 21 of the Code of Federal Regulations and/or the Declaration of Helsinki. The Principal Investigator is responsible for personally overseeing the treatment of all study patients. The Principal Investigator must assure that all study site personnel, including sub-investigators and other study staff members, adhere to the study protocol and all FDA/GCP/NCI regulations and guidelines regarding clinical trials both during and after study completion.

The Principal Investigator will be responsible for assuring that all the required data will be collected and entered onto the Case Report Forms. Periodically, monitoring visits will be conducted and the Principal Investigator will provide access to his/her original records to permit verification of proper entry of data. At the completion of the study, all case report forms will be reviewed by the Principal Investigator and will require his/her final signature to verify the accuracy of the data.

9.0 REFERENCES

1. Glare, P., et al., *A systematic review of physicians' survival predictions in terminally ill cancer patients.* BMJ, 2003. **327**(7408): p. 195-8.
2. Christakis, N.A. and E.B. Lamont, *Extent and determinants of error in doctors' prognoses in terminally ill patients: prospective cohort study.* BMJ, 2000. **320**(7233): p. 469-72.
3. Oken, M.M., et al., *Toxicity and response criteria of the Eastern Cooperative Oncology Group.* Am J Clin Oncol, 1982. **5**(6): p. 649-55.
4. Eastern Cooperative Oncology Group. *ECOG Performance Status*. March 21, 2015]; Available from: http://www.ecog.org/general/perf_stat.html.
5. Buccheri, G., D. Ferrigno, and M. Tamburini, *Karnofsky and ECOG performance status scoring in lung cancer: a prospective, longitudinal study of 536 patients from a single institution.* Eur J Cancer, 1996. **32A**(7): p. 1135-41.
6. Karnofsky, D. and J. Burchenal, *The clinical evaluation of chemotherapeutic agents in cancer*, in *Evaluation of chemptherapeutic agents*, C. McLeod, Editor., Columbia University Press: New York. p. 191-205.
7. Marechal, R., et al., *Prognostic factors and prognostic index for chemonaive and gemcitabine-refractory patients with advanced pancreatic cancer.* Oncology, 2007. **73**(1-2): p. 41-51.
8. Carson, K.A., et al., *Prognostic factors for survival in adult patients with recurrent glioma enrolled onto the new approaches to brain tumor therapy CNS consortium phase I and II clinical trials.* J Clin Oncol, 2007. **25**(18): p. 2601-6.
9. Sperduto, P.W., et al., *Summary report on the graded prognostic assessment: an accurate and facile diagnosis-specific tool to estimate survival for patients with brain metastases.* J Clin Oncol, 2012. **30**(4): p. 419-25.
10. Centers for Medicare and Medicaid.. *Oncology Care Model*. July 20, 2015]; Available from: http://innovation.cms.gov/initiatives/Oncology-Care/.
11. Fried, L.P., et al., *Frailty in older adults: evidence for a phenotype.* J Gerontol A Biol Sci Med Sci, 2001. **56**(3): p. M146-56.
12. Pal, S.K., V. Katheria, and A. Hurria, *Evaluating the older patient with cancer: understanding frailty and the geriatric assessment.* CA Cancer J Clin, 2010. **60**(2): p. 120-32.
13. Bassett, D.R., Jr., et al., *Pedometer-measured physical activity and health behaviors in U.S. adults.* Med Sci Sports Exerc, 2010. **42**(10): p. 1819-25.
14. Basch E, Snyder C, McNiff K et al. *Patient-reported outcome performance measures in oncology. J Oncol Pract 2014; 10: 209–211.*
15. Verger E, Salamero M, Conill C. Can Karnofsky performance status be transformed to the Eastern Cooperative Oncology Group scoring scale and vice versa? Eur J Cancer. 1992;28A(8-9):1328-30

10.0 APPENDICES

Appendix A: PROMIS Combined Survey Tool (Physical Function SF 20a; Sleep Disturbance SF 4a; Pain Interference SF 4a; Fatigue SF 8a)

In the past 7 days…

		Not at all	A little bit	Somewhat	Quite a bit	Very much
1	How much did pain interfere with your day to day activities?................................	☐	☐	☐	☐	☐
2	How much did pain interfere with work around the home?.....................................	☐	☐	☐	☐	☐
3	How much did pain interfere with your ability to participate in social activities?	☐	☐	☐	☐	☐
4	How much did pain interfere with your household chores?..................................	☐	☐	☐	☐	☐

		Without any difficulty	With a little difficulty	With some difficulty	With much difficulty	Unable to do
PFA11	Are you able to do chores such as vacuuming or yard work?..	☐ 5	☐ 4	☐ 3	☐ 2	☐ 1
PFA12	Are you able to push open a heavy door?	☐ 5	☐ 4	☐ 3	☐ 2	☐ 1
PFA16	Are you able to dress yourself, including tying shoelaces and doing buttons?	☐ 5	☐ 4	☐ 3	☐ 2	☐ 1
PFA34	Are you able to wash your back?	☐ 5	☐ 4	☐ 3	☐ 2	☐ 1
PFA38	Are you able to dry your back with a towel?	☐ 5	☐ 4	☐ 3	☐ 2	☐ 1
PFA51	Are you able to sit on the edge of a bed?	☐ 5	☐ 4	☐ 3	☐ 2	☐ 1
PFA55	Are you able to wash and dry your body?...........	☐ 5	☐ 4	☐ 3	☐ 2	☐ 1
PFA56	Are you able to get in and out of a car?	☐ 5	☐ 4	☐ 3	☐ 2	☐ 1
PFB19	Are you able to squeeze a new tube of toothpaste?..	☐ 5	☐ 4	☐ 3	☐ 2	☐ 1
PFB22	Are you able to hold a plate full of food?............	☐ 5	☐ 4	☐ 3	☐ 2	☐ 1
PFB24	Are you able to run a short distance, such as to catch a bus? …………………………………...	☐ 5	☐ 4	☐ 3	☐ 2	☐ 1

		Without any difficulty	With a little difficulty	With some difficulty	With much difficulty	Unable to do
PFB26	Are you able to shampoo your hair?......................	☐ 5	☐ 4	☐ 3	☐ 2	☐ 1
PFC45	Are you able to get on and off the toilet?	☐ 5	☐ 4	☐ 3	☐ 2	☐ 1
PFC46	Are you able to transfer from a bed to a chair and back?...	☐ 5	☐ 4	☐ 3	☐ 2	☐ 1

		Not at all	Very little	Somewhat	Quite a lot	Cannot do
PFA1	Does your health now limit you in doing vigorous activities, such as running, lifting heavy objects, participating in strenuous sports?	☐ 5	☐ 4	☐ 3	☐ 2	☐ 1
PFA3	Does your health now limit you in bending, kneeling, or stooping?	☐ 5	☐ 4	☐ 3	☐ 2	☐ 1
PFA5	Does your health now limit you in lifting or carrying groceries?	☐ 5	☐ 4	☐ 3	☐ 2	☐ 1
PFC12	Does your health now limit you in doing two hours of physical labor?...................................	☐ 5	☐ 4	☐ 3	☐ 2	☐ 1
PFC36	Does your health now limit you in walking more than a mile?	☐ 5	☐ 4	☐ 3	☐ 2	☐ 1
PFC37	Does your health now limit you in climbing one flight of stairs?...	☐ 5	☐ 4	☐ 3	☐ 2	☐ 1

In the past 7 days…

		Very poor	Poor	Fair	Good	Very good
1	My sleep quality was	☐	☐	☐	☐	☐

In the past 7 days…

		Not at all	A little bit	Somewhat	Quite a bit	Very much
2	My sleep was refreshing.	☐	☐	☐	☐	☐
3	I had a problem with my sleep..................	☐	☐	☐	☐	☐
4	I had difficulty falling asleep	☐	☐	☐	☐	☐

	**During the past 7 days…**	Not at all	A little bit	Somewhat	Quite a bit	Very much
HI7 1	I feel fatigued	☐ 1	☐ 2	☐ 3	☐ 4	☐ 5
AN3 2	I have trouble starting things because I am tired..	☐ 1	☐ 2	☐ 3	☐ 4	☐ 5
	**In the past 7 days…**					
FATEXP41 3	How run-down did you feel on average? ...	☐ 1	☐ 2	☐ 3	☐ 4	☐ 5
FATEXP40 4	How fatigued were you on average?	☐ 1	☐ 2	☐ 3	☐ 4	☐ 5
FATEXP35 5	How much were you bothered by your fatigue on average?....................................	☐ 1	☐ 2	☐ 3	☐ 4	☐ 5
FATIMP49 6	To what degree did your fatigue interfere with your physical functioning?	☐ 1	☐ 2	☐ 3	☐ 4	☐ 5

	**In the past 7 days…**	Never	Rarely	Sometimes	Often	Always
FATIMP3 7	How often did you have to push yourself to get things done because of your fatigue?	☐ 1	☐ 2	☐ 3	☐ 4	☐ 5
FATIMP16 8	How often did you have trouble finishing things because of your fatigue?	☐ 1	☐ 2	☐ 3	☐ 4	☐ 5

	**In the past 7 days...**	Never	Rarely	Sometimes	Often	Always
EDDEP04 1	I felt worthless	☐ 1	☐ 2	☐ 3	☐ 4	☐ 5
EDDEP06 2	I felt helpless	☐ 1	☐ 2	☐ 3	☐ 4	☐ 5
EDDEP29 3	I felt depressed	☐ 1	☐ 2	☐ 3	☐ 4	☐ 5
EDDEP41 4	I felt hopeless	☐ 1	☐ 2	☐ 3	☐ 4	☐ 5

Appendix B: Additional Questions Added to Final Weekly Patient Self-Assessment

*Which **one** of the following categories do you believe best represents your level of functional capacity over the past week (please select the one best answer):*

Mark One Category	Description
	Normal activity. Fully active, able to carry on all pre-cancer diagnosis performance without restriction.
	Symptoms but ambulatory. Restricted in physically strenuous activity, but ambulatory and able to carry out work of a light or sedentary nature (e.g., light housework, office work).
	In bed <50% of the time. Ambulatory and capable of all self-care, but unable to carry out any work activityies. Up and about more than 50% of waking hours.
	In bed >50% of the time. Capable of only limited self-care, confined to bed or chair more that 50% of waking hours.
	100% bedridden. Completely disabled. Cannot carry on any self care. Totally confined to bed or chair.

*Now, with a different group of categories, which **one** of the following categories do you believe best represents your level of functional capacity over the past week (please select the one best answer):*

Mark One Category	Description
	Normal; no complaints; no evidence of disease
	Able to carry out normal activity; minor signs or symptoms of disease
	Normal activity with effort; some signs or symptoms of disease
	Cares for self; unable to carry on normal activity or to do active work
	Requires occasional assistance but is able to care for most personal needs
	Requires considerable assistance and frequent medical care
	Disabled; requires special care and assistance
	Severely disabled; hospitalization is indicated, although death not imminent
	Very sick; hospitalization necessary; active support treatment is necessary
	Moribund; fatal process

Appendix C: Exit Interview

Follow-up Intervention Script- ECOG-Fitbit Sensor Exploratory Study

We want to thank you for your participation in the study and for coming in today to meet with us. This interview will take approximately 60 minutes, and we will ask you a number of open-ended questions about your thoughts and feelings about your experience wearing the sensors and answering the online assessments. There are no 'right' or 'wrong' answers to any of these questions: we want to hear about your experience and listen to your opinion, in your own words.

General "Think Aloud" probe

- *When you think about being in the study during the past two weeks, what is the first thing that comes to your mind?*

Wearing the Sensors

- *Tell us about your experience wearing the Fitbit Device.*
- *Did you have any problems with the sensors? If so, what problems did you have?*
- *Do you think there are places on your body that you would have preferred to wear the sensors? Are there places that would have been easier for you to wear the sensors? Where?*
- *Did the sensors interfere with any of your usual activities? If so, how?*
- *Tell us about your experience putting on and removing the sensors.*
- *Tell us about your experience charging the sensor.*
- *Tell us about your experience syncing the sensor with your phone.*
- *Did you ever have to remove the sensors because they were uncomfortable?*
- *Were there certain times of the day you had trouble wearing the sensors?*
- *Did you ever forget to wear the sensor after removing? How often? How long?*
- *Did you ever view your activity log on your smartphone? If so, how often? Do you think that viewing the information changed your behavior? If so, how?*
- *Do you have any thoughts about how to improve the sensors?*
- *Did anyone ever ask about your sensors? What did they ask? How did you respond? How did you feel about people noticing your sensors? How did you feel about people asking about your sensors?*
- *In your opinion, would it be useful to share the sensor information with your doctor?*

Online Symptom Reporting

- *Did you have any difficulty filling out the online questionnaires? If so, tell us about it.*
- *Would you have preferred to use a smartphone or tablet device to complete the assessments? Why or why not?*
- *What do you think about how often you were asked to complete the online questionnaires?*
- *What did you think about the length of the online questionnaires?*
    - *(IF THEY WERE TOO LONG): In your opinion, what would be an acceptable length for a survey?*
- *What did you think about the frequency of the online questionnaires?*
    - *(IF TOO OFTEN): How often would be an acceptable frequency?*
- *Would you liked to see a report of your sensor data? How do you imagine this information being displayed?*
- *In your opinion, would it be useful to share the questionnaire data with your doctor?*

This is all the questions we have. Is there anything we didn't mention that you would like to discuss? Thank you again for your participation in this study.

Appendix D- Measures of Frailty

Table 1. Operationalizing a Phenotype of Frailty

A. *Characteristics of Frailty*	B. *Cardiovascular Health Study Measure**
Shrinking: Weight loss (unintentional) Sarcopenia (loss of muscle mass)	Baseline: >10 lbs lost unintentionally in prior year
Weakness	Grip strength: lowest 20% (by gender, body mass index)
Poor endurance; Exhaustion	"Exhaustion" (self-report)
Slowness	Walking time/15 feet: slowest 20% (by gender, height)
Low activity	Kcals/week: lowest 20% males: <383 Kcals/week females: <270 Kcals/week
	C. *Presence of Frailty*
	Positive for frailty phenotype: ≥3 criteria present
	Intermediate or prefrail: 1 or 2 criteria present

Table from Fried et al. 2001[11]

Protocol for frailty criteria*:

D1. Weight loss: Weigh patient and assess for weight loss. Someone who is frail may have unintentional weight loss of ≥ 10 pounds in the prior year.

D2. Gait speed: Time a patient's walk for slowness. Someone who is frail has a decreased walking time as defined by a timed 15-foot walk test (5 meters). The time is adjusted for gender and standing height. Men with a height of <173 cm and women with a height <159 cm who walked 15 feet in >7 sec are considered frail; men >173 cm and women >159 cm who walked 15 feet in >6 sec are considered frail.

D3. Weakness: Weakness is established when there is decreased grip strength measured by a dynamometer with the value adjusted for gender and body mass index (BMI). Men with a BMI <24 are considered frail if the grip strength (kg) is <29, for a BMI of 24.1-28, a man is frail if <30, for a BMI >28 a man is frail if <32. For women, a BMI of <23 is considered frail if the grip strength (kg) is <17, a BMI 23.1-26 is considered frail if <17.3, a BMI of 26.1-29 is considered frail if <18, and a BMI >29 is considered frail if <21.

D4. Physical Activity: Determine if the patient has a low physical activity level. This is established by a weighted score of kilocalories expended per week measured by the Minnesota Leisure Time Activity Questionnaire. The questionnaire asks about activities like daily living, sports and hobbies. Frailty is present when males use <383kcal/week, and females <270 kcal/week.

D5. Frailty Phenotype: Score will be calculated based on presence of each of these characteristics where presence of 1-2 criteria are categorized as prefail, and >=3 critiera categorized as frail.

* Derived from Frailty score in [11]
